# Visible-light-assisted photocatalytic removal of methylene blue over ternary ZnO/Zn_2_SnO_4_/rGO heterostructures: structural, optical, and charge-transport behavior

**DOI:** 10.1039/d6ra05978c

**Published:** 2026-09-03

**Authors:** Van Tuan Pham, Phi Hung Hoang, Thi Tam Hien Vu, Tien Ha Le, Ngoc Khiem Tran

**Affiliations:** a School of Materials Science and Engineering, Hanoi University of Science and Technology No. 1, Dai Co Viet Hanoi Vietnam tuan.phamvan@hust.edu.vn; b Institute of Science and Technology, TNU-University of Sciences Phan Dinh Phung Thai Nguyen 250000 Vietnam halt@tnus.edu.vn

## Abstract

Interfacial engineering of semiconductor heterostructures with conductive carbon materials offers a promising strategy for developing visible-light-assisted photocatalysts. In this study, ZnO/Zn_2_SnO_4_/rGO (ZSO/rGO) ternary composites with different rGO contents were synthesized *via* a hydrothermal method. XRD and Raman analyses confirmed the coexistence of hexagonal ZnO, cubic spinel Zn_2_SnO_4_, and rGO, while electron microscopy revealed oxide particles distributed in close contact with thin rGO sheets. XPS further indicated changes in the surface chemical and electronic environments after Zn_2_SnO_4_ and rGO incorporation. The optimized ZSO2.5 composite, containing 2.5 wt% rGO, exhibited the lowest photoluminescence intensity and a transient photocurrent response nearly twice that of binary ZnO/Zn_2_SnO_4_ (ZSO), supporting reduced radiative recombination and improved photoinduced carrier transport. ZSO2.5 achieved the highest methylene blue removal efficiency of 87.94% after 120 min of visible-light irradiation, with an apparent rate constant of 0.01764 min^−1^. Scavenger experiments indicated that ˙O_2_^−^ made the most substantial contribution among the investigated reactive species, followed by ˙OH and h^+^. The enhanced performance is attributed to the combined effects of the ZnO/Zn_2_SnO_4_ interface, rGO-assisted light absorption and adsorption, and conductive pathways for carrier transport. However, the available results do not conclusively distinguish between Type-II and Z-scheme-like charge-transfer pathways. Because transformation products and mineralization were not directly analyzed, the observed process is described as methylene blue removal and decolorization rather than complete degradation. These findings demonstrate the potential of ZSO/rGO composites for visible-light-assisted water treatment.

## Abstract 2

Interfacial engineering of semiconductor heterostructures with conductive carbon materials offers a promising strategy for developing visible-light-assisted photocatalysts.

## Graphical abstract

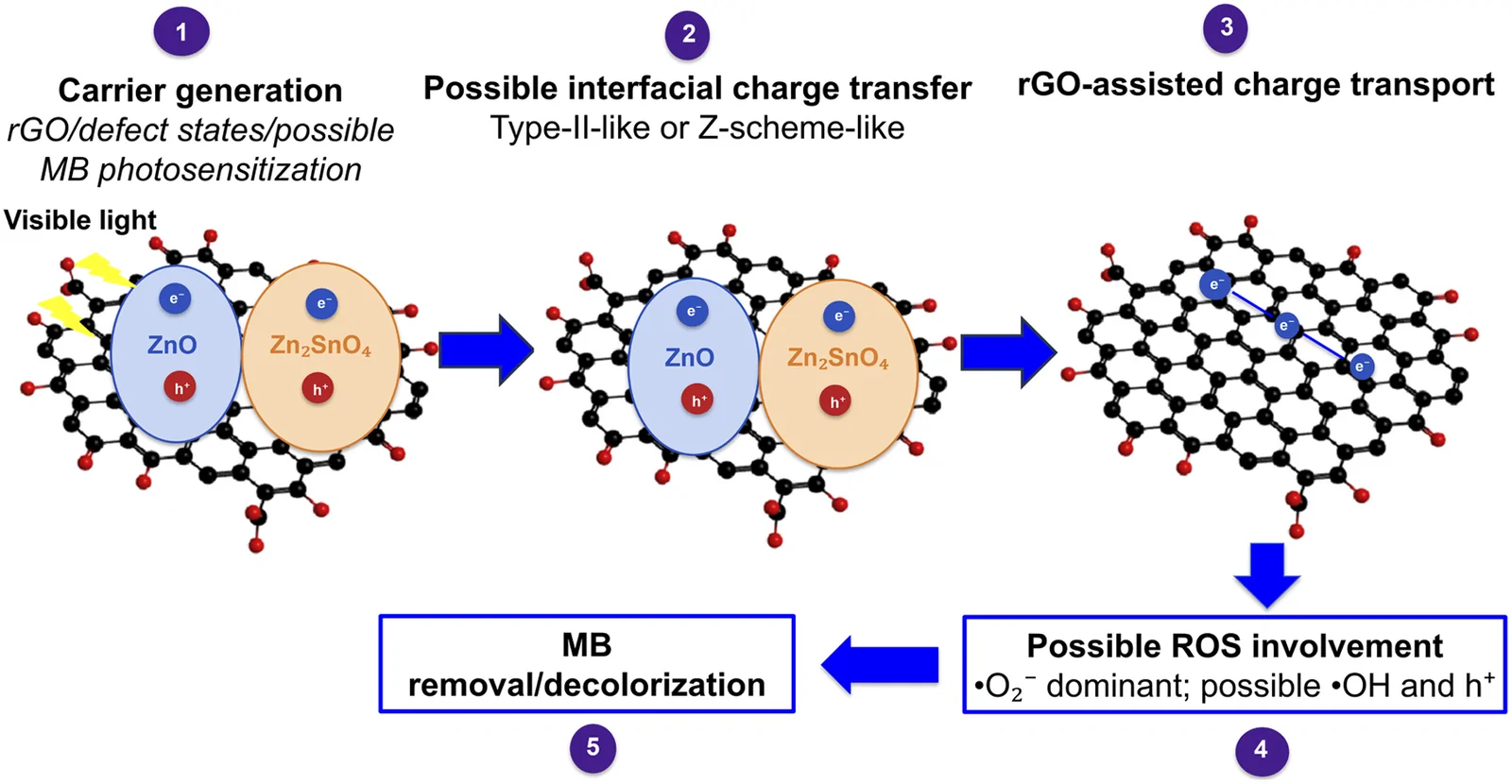

## 1. Introduction

Environmental water pollution has become a major global challenge because of rapid industrialization, urbanization, and population growth.^1–4^ The discharge of insufficiently treated domestic, industrial, and agricultural wastewater has adversely affected aquatic ecosystems and groundwater resources.^1,2,4^ Among the contaminants of concern, recalcitrant organic compounds, including dyes such as methylene blue (MB) and rhodamine B (RhB), are particularly problematic because of their chemical stability, persistence, and potential adverse effects on human health and aquatic organisms.^2–5^ Recent reviews have further highlighted the potential of spinel-based photocatalysts and their carbon-containing composites for visible-light-assisted MB removal, while emphasizing that adsorption, reactive-species formation, pollutant transformation, and mineralization should be evaluated separately.^6^

Conventional treatment methods, including adsorption, extraction, and electrochemical processes, have been widely employed to remove organic pollutants from water. However, these methods may involve pollutant transfer rather than destruction, relatively high operating costs, or the generation of secondary waste.^1,2,4^ Semiconductor photocatalysis has therefore attracted considerable interest as an advanced oxidation process because of its mild operating conditions, potential utilization of solar energy, and environmental compatibility. Upon light irradiation, semiconductor photocatalysts generate electron–hole pairs, which can subsequently produce reactive oxygen species such as ˙OH and ˙O_2_^−^. These species can oxidatively transform organic pollutants and, under suitable conditions, promote their mineralization into CO_2_, H_2_O, and inorganic products.^1,4,7^

The performance of many single-phase photocatalysts is limited by poor visible-light absorption and rapid recombination of photogenerated charge carriers.^1–4^ ZnO has been extensively investigated because of its chemical stability, low toxicity, availability, and relatively low fabrication cost.^1,2,8^ Nevertheless, its wide band gap restricts its intrinsic absorption mainly to the ultraviolet region, while rapid carrier recombination and possible photocorrosion limit its practical photocatalytic efficiency.^2,8,9^

Constructing ZnO-based heterostructures with other semiconductor oxides is an effective strategy for modifying interfacial carrier behavior and improving photocatalytic performance.^10–12^ Zn_2_SnO_4_ is an n-type spinel semiconductor with good chemical and photochemical stability, electrical conductivity, and a wide band gap of approximately 3.6 eV.^12–14^ Coupling ZnO, with a band gap of approximately 3.2–3.3 eV, and Zn_2_SnO_4_ can create an oxide–oxide interface that may facilitate interfacial charge separation and transport.^12,13,15^ However, because both phases are wide-band-gap semiconductors, binary ZnO/Zn_2_SnO_4_ (ZSO) still exhibits limited intrinsic visible-light absorption and may not completely suppress charge recombination.^12,13^ Moreover, distinguishing between conventional Type-II and Z-scheme-like charge-transfer pathways generally requires complementary experimental evidence capable of determining the band alignment and direction of interfacial carrier migration.^16^

Ternary composites such as ZnO/SnO_2_/rGO, Zn_2_SnO_4_/SnO_2_/g-C_3_N_4_, and ZnO–SnO_2_–Zn_2_SnO_4_ have shown improved photocatalytic behavior through the combination of multiple interfaces and conductive or photoactive components.^10,17–21^ However, ZnO/Zn_2_SnO_4_/rGO (ZSO/rGO) ternary composites remain comparatively underexplored. rGO possesses broadband optical absorption, a large accessible surface area, and an extended π-conjugated network that can support pollutant adsorption and interfacial carrier transport.^8,17,22^ Nevertheless, the effects of rGO content on the structural, optical, photoelectrochemical, and photocatalytic properties of ZSO/rGO composites have not been systematically established. In particular, the relationship between rGO loading, interfacial carrier behavior, and photocatalytic performance requires further clarification.

In this study, ZSO/rGO ternary composites with different rGO contents were synthesized *via* a hydrothermal method. The effects of rGO loading on their phase composition, morphology, surface chemical states, optical response, radiative recombination, transient photocurrent response, and visible-light-assisted MB removal were systematically examined. Possible interfacial carrier-transfer pathways are discussed cautiously based on the combined experimental observations and literature data. This work establishes a structure–property–performance relationship for ZSO/rGO composites and provides insights into the design of oxide/rGO photocatalysts for visible-light-assisted water treatment.

## 2. Experimental

### 2.1. Chemicals

All chemicals were of analytical grade and used without further purification. Zinc chloride (ZnCl_2_, ≥98%, Merck, Germany), tin(iv) chloride pentahydrate (SnCl_4_·5H_2_O, ≥98%, Sigma-Aldrich, USA), sodium hydroxide (NaOH, ≥98%, Xilong Chemical Co., China), absolute ethanol (C_2_H_5_OH, 99.9%, Merck, Germany), and graphene oxide (GO, ≥98%, Sigma-Aldrich, USA) were used as received. Deionized water was used for solution preparation and sample washing. Unless otherwise specified, solution preparation and precipitation were conducted at room temperature.

### 2.2. Material synthesis

#### 2.2.1. Synthesis of the ZnO/rGO nanocomposite

The binary ZnO/rGO nanocomposite was synthesized *via* a one-step hydrothermal method. First, 10.0 g of ZnCl_2_ was dissolved in 20 mL of deionized water under magnetic stirring at 600 rpm for 30 min. Separately, 5.9 g of NaOH was dissolved in 20 mL of deionized water. The ZnCl_2_ solution was then added dropwise to the NaOH solution under continuous stirring at room temperature. The resulting suspension was aged for 30 min to promote Zn(OH)_2_ precipitation.

Subsequently, 20 mL of absolute ethanol and 0.06 g of GO, corresponding to approximately 1.0 wt% relative to the theoretical ZnO yield, were added to the suspension. The mixture was stirred for an additional 20 min to promote GO dispersion and then transferred to a 100 mL Teflon-lined stainless-steel autoclave. Hydrothermal treatment was performed at 180 °C for 24 h, during which Zn(OH)_2_ was converted into crystalline ZnO and GO underwent partial *in situ* reduction to rGO.

After the autoclave had cooled naturally to room temperature, the product was collected by centrifugation at 4000 rpm for 10 min. The precipitate was washed repeatedly with deionized water and absolute ethanol until the supernatant reached approximately neutral pH and was subsequently dried at 70 °C for 24 h. The resulting material was denoted as ZnO/rGO.

#### 2.2.2. Synthesis of the ZSO heterostructure

The binary ZSO heterostructure was synthesized *via* a one-step hydrothermal method using ZnCl_2_ and SnCl_4_·5H_2_O as the Zn^2+^ and Sn^4+^ precursors, respectively. The precursors were separately dissolved in deionized water and then mixed in proportions corresponding to a nominal ZnO : Zn_2_SnO_4_ mass ratio of 8 : 2. A 2 M NaOH solution was added dropwise under continuous magnetic stirring until the pH reached 9–10, promoting the formation of zinc- and tin-containing hydroxide intermediates.

The resulting suspension was transferred to a Teflon-lined stainless-steel autoclave and hydrothermally treated at 180 °C for 24 h. During this process, the hydroxide intermediates were converted into crystalline ZnO and Zn_2_SnO_4_. After the autoclave had cooled naturally to room temperature, the product was collected by filtration, washed repeatedly with deionized water and absolute ethanol to remove residual ions, and dried at 80 °C for 12 h. The resulting binary composite was denoted as ZSO and used as the reference material for comparison with the rGO-containing ternary composites.

#### 2.2.3. Synthesis of ZSO/rGO nanocomposites

The ternary ZSO/rGO nanocomposites were synthesized *via* a one-step hydrothermal method by incorporating graphene oxide (GO) into the binary ZSO system, followed by its partial *in situ* reduction. Briefly, GO was added at nominal loadings of 1.0, 2.5, and 5.0 wt% relative to the total nominal mass of the metal oxides and dispersed in deionized water by probe ultrasonication for 30 min to obtain a homogeneous suspension. Meanwhile, aqueous precursor solutions containing Zn^2+^ and Sn^4+^ ions were prepared separately and mixed to achieve a nominal ZnO : Zn_2_SnO_4_ mass ratio of 8 : 2. The GO suspension was then introduced into the mixed precursor solution under continuous magnetic stirring, followed by the dropwise addition of 2 M NaOH until the pH reached 9–10, thereby promoting the co-precipitation of Zn(OH)_2_ and Sn(OH)_4_ onto the GO sheets. The resulting suspension was transferred to a Teflon-lined stainless-steel autoclave and hydrothermally treated at 180 °C for 24 h, during which the hydroxide precursors were converted into crystalline ZnO and Zn_2_SnO_4_ nanoparticles, while GO underwent partial *in situ* reduction to rGO. The obtained precipitate was collected by filtration, thoroughly washed with deionized water and absolute ethanol, and dried at 80 °C for 12 h. The synthesized samples were designated according to their nominal oxide compositions and rGO loadings, as summarized in Table 1. The rGO loading was calculated relative to the total nominal mass of the metal oxides and was not included in the normalization of the ZnO : Zn_2_SnO_4_ oxide ratio.

**Table 1:** Sample codes, nominal oxide compositions, and nominal rGO loadings of the synthesized materials

Sample code	Nominal oxide composition (wt%)	Nominal rGO loading (wt% relative to total oxide mass)
ZnO	ZnO = 100	0.0
ZnO/rGO	ZnO = 100	1.0
ZSO	ZnO : Zn_2_SnO_4_ = 80 : 20	0.0
ZSO1.0	ZnO : Zn_2_SnO_4_ = 80 : 20	1.0
ZSO2.5	ZnO : Zn_2_SnO_4_ = 80 : 20	2.5
ZSO5.0	ZnO : Zn_2_SnO_4_ = 80 : 20	5.0

### 2.3. Material characterization

The crystal structures of the synthesized materials were characterized by X-ray diffraction (XRD) using a diffractometer equipped with Cu Kα radiation (*λ* = 1.5406 Å) over a 2*θ* range of 10–80°. The morphology and microstructure of the samples were examined by field-emission scanning electron microscopy (FESEM), while their elemental composition and spatial distribution were analyzed using energy-dispersive X-ray spectroscopy (EDX). Transmission electron microscopy (TEM) was employed to further investigate the particle morphology, dispersion, and interfacial contact between the oxide particles and graphene-derived sheets. TEM observations were carried out using a JEM-1400 Flash transmission electron microscope (JEOL, Japan).

Fourier-transform infrared (FTIR) spectra were recorded in the wavenumber range of 400–4000 cm^−1^ using the KBr pellet method at room temperature. Raman scattering spectra were acquired using a high-resolution Raman spectrometer equipped with a 532 nm solid-state excitation laser. The laser power was maintained at a sufficiently low level to minimize laser-induced heating of the samples. Spectra were collected over the Raman shift range of 100–2000 cm^−1^.

Steady-state photoluminescence (PL) spectra were recorded at room temperature using a NanoLog spectrofluorometer (HORIBA Jobin Yvon) equipped with a 450 W xenon lamp, with an excitation wavelength of 325 nm, to investigate the radiative recombination behavior of photogenerated charge carriers. Optical absorption spectra were measured using a Jasco V-650 UV-vis spectrophotometer over the wavelength range of 200–800 nm. Prior to measurement, the composite powders were dispersed in deionized water by probe ultrasonication for 30 min to obtain homogeneous suspensions. The suspensions were analyzed in quartz cuvettes using deionized water as the reference. The apparent optical transition energies were estimated from the measured absorbance data using a Tauc-type analysis.

The surface chemical composition and oxidation states of the constituent elements were investigated by X-ray photoelectron spectroscopy (XPS). High-resolution spectra were collected for the Zn 2p, Sn 3d, O 1s, and C 1s regions for subsequent analysis.

### 2.4. Photocatalytic activity evaluation

The photocatalytic activity of the synthesized samples was evaluated through the removal/decolorization of methylene blue (MB) under visible-light irradiation. In a typical experiment, 20.0 mg of photocatalyst was dispersed in 60 mL of an aqueous MB solution (1.0 × 10^−5^ M). The initial pH of the MB solution was adjusted to 7.0. Prior to irradiation, the suspension was magnetically stirred in the dark for 60 min to establish adsorption–desorption equilibrium. Visible-light irradiation was provided by a commercial white LED floodlight (30 W, 6500 K) positioned 10 cm above the reaction vessel. At predetermined time intervals, 3 mL aliquots were withdrawn and immediately centrifuged to separate the photocatalyst. The residual MB concentration was estimated from its absorbance at 664 nm using a Jasco V-650 UV-vis spectrophotometer. The MB removal efficiency (*η*) was calculated according to:*η* (%) = [(*C*_0_ − *C*_*t*_)/*C*_0_] × 100where *C*_0_ is the MB concentration at the beginning of visible-light irradiation after dark adsorption–desorption equilibrium, and *C*_*t*_ is the MB concentration at irradiation time *t*. The photocatalytic reaction kinetics were evaluated using an apparent pseudo-first-order kinetic model:ln(*C*_0_/*C*_*t*_) = *k*_app_*t*where *k*_app_ (min^−1^) is the apparent reaction rate constant.

Radical trapping experiments were conducted using ZSO2.5 to evaluate the relative contributions of reactive species to the photocatalytic removal of MB. Isopropanol (IPA), benzoquinone (BQ), and ethylenediaminetetraacetic acid (EDTA) were employed as scavengers for hydroxyl radicals (˙OH), superoxide radicals (˙O_2_^−^), and photogenerated holes (h^+^), respectively. In addition, reusability tests were conducted over three consecutive photocatalytic MB removal cycles using ZSO2.5 to evaluate its short-term operational stability. The changes in MB concentration during the radical trapping and cycling experiments were monitored using a Jasco V-650 UV-vis spectrophotometer by recording the temporal variation in the characteristic MB absorbance at 664 nm.

### 2.5. Photoelectrochemical measurements

Photoelectrochemical (PEC) measurements were performed using a Zahner Zennium Pro electrochemical workstation coupled with a Zahner PP212 external potentiostat (Zahner-Elektrik GmbH, Germany). The working electrodes were prepared by coating the synthesized photocatalysts onto indium tin oxide (ITO) glass substrates. All measurements were carried out at room temperature in a conventional three-electrode configuration consisting of the catalyst-coated ITO as the working electrode, a platinum wire as the counter electrode, and an Ag/AgCl (saturated KCl) electrode as the reference electrode. Transient photocurrent measurements were conducted under chopped visible-light irradiation using a Zahner CIMPS-QE/IPCE system equipped with a TLS03 tunable light source. The photocurrent responses (*I*–*t* curves) were recorded at an applied potential of 0.2 V *versus* Ag/AgCl under periodic light on/off cycles.

## 3. Results and discussion

### 3.1. X-ray diffraction (XRD) analysis

#### 3.1.1. Crystal phase formation of the samples

Fig. 1 presents the XRD patterns of pristine ZnO, ZnO/rGO, binary ZSO, and ternary ZSO/rGO composites containing 1.0, 2.5, and 5.0 wt% rGO. The results show the coexistence of ZnO and Zn_2_SnO_4_ crystalline phases in the binary and ternary samples, with no detectable crystalline impurity phases. The diffraction peaks of pristine ZnO located at 2*θ* ≈ 31.68°, 34.43°, 36.09°, 47.44°, 56.53°, 62.98°, 66.32°, 67.88°, and 69.07° are indexed to the (100), (002), (101), (102), (110), (103), (200), (112), and (201) planes, respectively, of hexagonal wurtzite ZnO (JCPDS No. 36-1451), indicating good crystallinity.^8,22^ No distinct diffraction peak associated with rGO is observed near 2*θ* ≈ 26°, which may be attributed to its low content, limited graphitic ordering, and weak X-ray scattering intensity.^22,23^

**Fig. 1:**
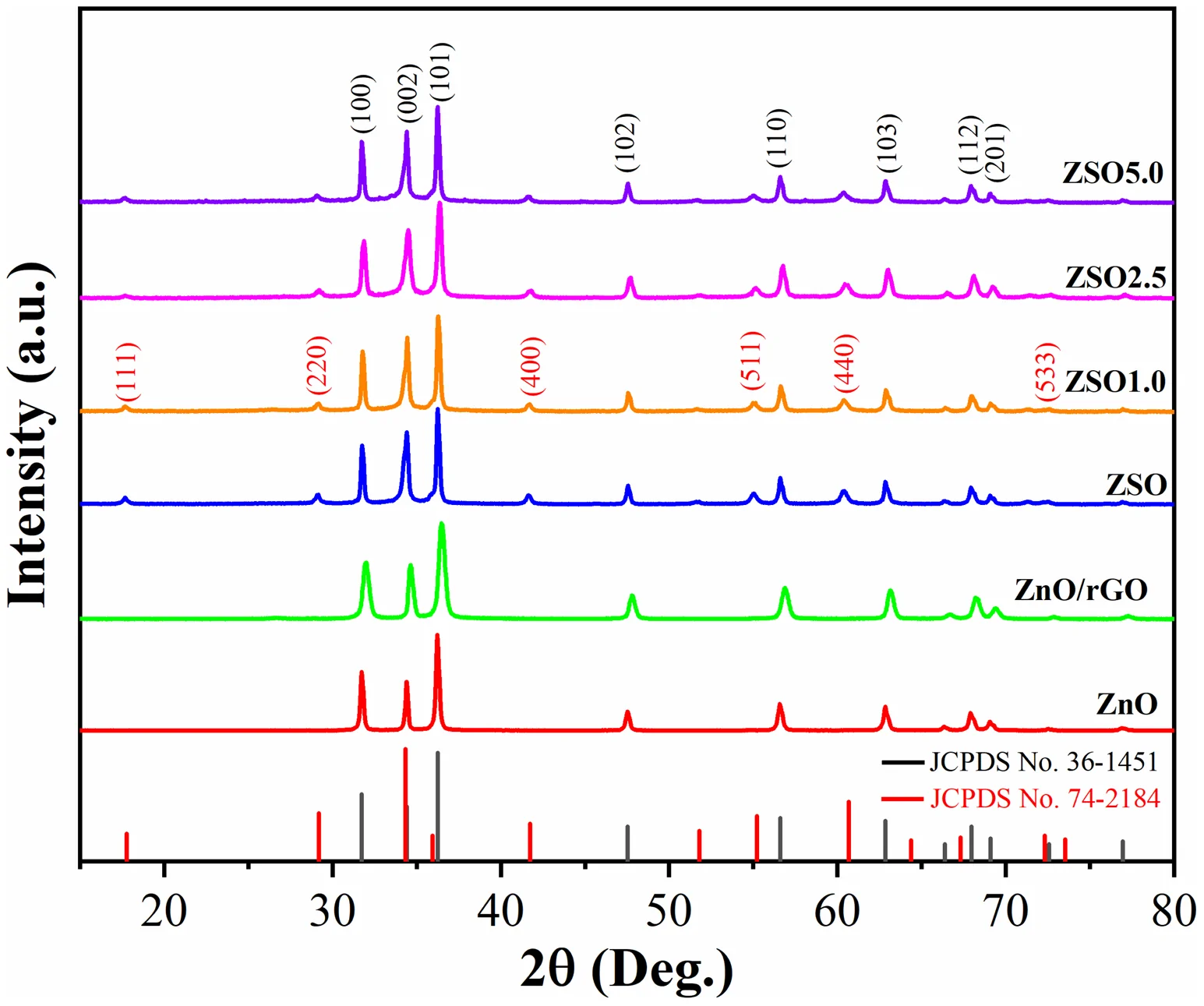
XRD patterns of ZnO, ZnO/rGO, ZSO, and ZSO/rGO composites with different rGO contents. Reference patterns of hexagonal ZnO and cubic Zn_2_SnO_4_ are shown at the bottom.

For the ZSO and ZSO/rGO composites, additional diffraction peaks appear at 2*θ* ≈ 17.71°, 29.06°, 41.59°, 51.63°, 55.10°, and 60.47°, corresponding to the (111), (220), (400), (422), (511), and (440) planes, respectively, of cubic spinel Zn_2_SnO_4_ (JCPDS No. 74-2184).^12,13^ No diffraction peaks attributable to crystalline SnO_2_ or other secondary phases are detected within the detection limit of XRD. These observations indicate the formation of Zn_2_SnO_4_ alongside ZnO and support the successful preparation of the binary ZnO/Zn_2_SnO_4_ composite.

After rGO incorporation, no appreciable shifts in the characteristic peak positions of ZnO and Zn_2_SnO_4_ are observed, although slight variations in peak width and relative intensity occur.^8,23^ This indicates that rGO does not alter the fundamental crystal structures of the two oxide phases, while its influence on ZnO crystallite growth is discussed quantitatively in Section 3.1.2. The continued absence of a distinct rGO diffraction peak is consistent with its low loading and limited graphitic ordering. However, XRD alone cannot confirm the dispersion or restacking behavior of rGO; these features should be evaluated using Raman spectroscopy and electron microscopy. Overall, the XRD results demonstrate that the ZnO and Zn_2_SnO_4_ crystalline phases are retained after rGO incorporation, providing a structural basis for the formation of oxide–oxide and oxide–rGO interfaces.

#### 3.1.2. Influence of rGO on the crystallization and crystallite size of ZnO

The XRD results indicate that rGO incorporation affects the crystallization behavior and crystallite size of ZnO in the ZSO/rGO heterostructure. Although no appreciable shifts in the ZnO peak positions are observed, the diffraction peaks become slightly broader and their relative intensities decrease after rGO incorporation. These changes are consistent with a reduction in the coherent crystallite size of ZnO. During hydrothermal synthesis, oxygen-containing functional groups and defect sites on the graphene-derived sheets may provide heterogeneous nucleation sites and restrict the growth or coalescence of ZnO crystallites.^22,24–26^

The ZnO crystallite sizes were estimated from the three most intense diffraction peaks, corresponding to the (100), (002), and (101) planes, using the Scherrer equation:^24,25^*D* = *Kλ*/(*β* cos *θ*)where *D* is the crystallite size (nm), *K* = 0.9 is the shape factor, *λ* = 1.5406 Å is the X-ray wavelength, *β* is the full width at half maximum of the diffraction peak expressed in radians, and *θ* is the corresponding Bragg angle. The crystallite sizes calculated from the three reflections were averaged to obtain the values reported in Table 2.

**Table 2:** ZnO crystallite sizes calculated from the (100), (002), and (101) diffraction peaks using the Scherrer equation

Samples	*D*_(100)_ (nm)	*D*_(002)_ (nm)	*D*_(101)_ (nm)	*D*_avg_ (nm)
ZnO	38.16	43.43	35.56	39.05
ZnO/rGO	19.45	25.85	19.64	21.65
ZSO	42.80	23.38	38.24	34.81
ZSO1.0	40.41	23.92	36.92	33.75
ZSO2.5	30.53	19.97	27.99	26.16
ZSO5.0	41.95	23.58	37.72	34.42

Pristine ZnO exhibits an average crystallite size of 39.05 nm, whereas ZnO/rGO shows a markedly lower value of 21.65 nm. This reduction supports the proposed role of the graphene-derived sheets in restricting ZnO crystallite growth. The average ZnO crystallite size in binary ZSO is 34.81 nm. After the incorporation of 1.0 wt% rGO, the value decreases slightly to 33.75 nm, followed by a more pronounced reduction to 26.16 nm for ZSO2.5. These results indicate that an appropriate rGO content can restrict the growth and coalescence of ZnO crystallites under the present synthesis conditions.^22,24,26^

When the rGO content is increased to 5.0 wt%, the average ZnO crystallite size increases to 34.42 nm, approaching that of binary ZSO. One possible explanation is that excessive rGO loading promotes partial sheet restacking or aggregation, reducing the effective oxide rGO interfacial area and its ability to restrict crystallite growth.^22,25,26^ However, this proposed explanation cannot be confirmed by XRD alone.

Overall, ZSO2.5 exhibits the smallest average ZnO crystallite size among the ternary composites, indicating the most effective regulation of ZnO crystallization at 2.5 wt% rGO. The smaller crystallites may increase the available interfacial area and density of surface-active sites. Nevertheless, their contribution to charge separation and photocatalytic performance should be interpreted together with the morphological, optical, photoelectrochemical, and photocatalytic results.

#### 3.1.3. Proposed formation mechanism of the ZSO/rGO heterostructure

Based on the synthesis conditions and phase composition identified by XRD, a possible formation mechanism for the ZSO/rGO heterostructure is proposed. During hydrothermal synthesis, Zn^2+^ and Sn^4+^ species undergo hydrolysis to form zinc- and tin-containing hydroxide intermediates. The process can be represented by the following simplified reactions:Zn^2+^ + 2OH^−^ → Zn(OH)_2_Sn^4+^ + 4OH^−^ → Sn(OH)_4_Zn(OH)_2_ → ZnO + H_2_O2Zn(OH)_2_ + Sn(OH)_4_ → Zn_2_SnO_4_ + 4H_2_O

For the nominal ZnO/Zn_2_SnO_4_ mass ratio of 8 : 2, only part of the zinc-containing precursor reacts with the tin-containing species to form Zn_2_SnO_4_, whereas the remaining zinc precursor crystallizes as ZnO. The coexistence of hexagonal ZnO and cubic Zn_2_SnO_4_ is supported by the XRD results and is consistent with previous studies on ZnO/Zn_2_SnO_4_ composites.^11,27^

GO undergoes partial reduction under hydrothermal conditions through the removal of oxygen-containing functional groups, resulting in rGO with improved electrical conductivity.^22,24^ Residual oxygen-containing groups and defect sites on the graphene-derived sheets may coordinate with Zn^2+^ and Sn^4+^ species and provide heterogeneous nucleation sites for oxide formation.^24,26^ Consequently, ZnO and Zn_2_SnO_4_ may nucleate and grow in contact with the rGO sheets, promoting the formation of oxide–oxide and oxide–carbon interfaces. This interpretation is consistent with the reduction in ZnO crystallite size observed at appropriate rGO loadings and is further evaluated using Raman spectroscopy, electron microscopy, and XPS.

The resulting material can therefore be described as a ternary composite comprising ZnO and Zn_2_SnO_4_ crystalline domains coupled with rGO sheets. The oxide–rGO interfaces may provide pathways for carrier transport. In particular, rGO may act as a conductive charge-transport mediator because of its extended π-conjugated structure.^17,22,25^ However, neither the direction of charge transfer nor the band alignment can be established from XRD. These aspects are therefore discussed cautiously based on the combined optical, XPS, PL, photocurrent, and reactive-species results.

Overall, the hydrothermal process enables the concurrent formation of ZnO and Zn_2_SnO_4_ in the presence of partially reduced graphene sheets. The resulting oxide–oxide and oxide–rGO interfaces provide a structural basis for the carrier-transport and photocatalytic behavior examined in the subsequent sections.

### 3.2. Raman analysis

Raman spectroscopy was employed to examine the vibrational characteristics of the prepared samples, and the corresponding spectra are shown in Fig. 2. The simultaneous observation of Raman features associated with ZnO, Zn_2_SnO_4_, and rGO supports the coexistence of these components in the ternary composites.

**Fig. 2:**
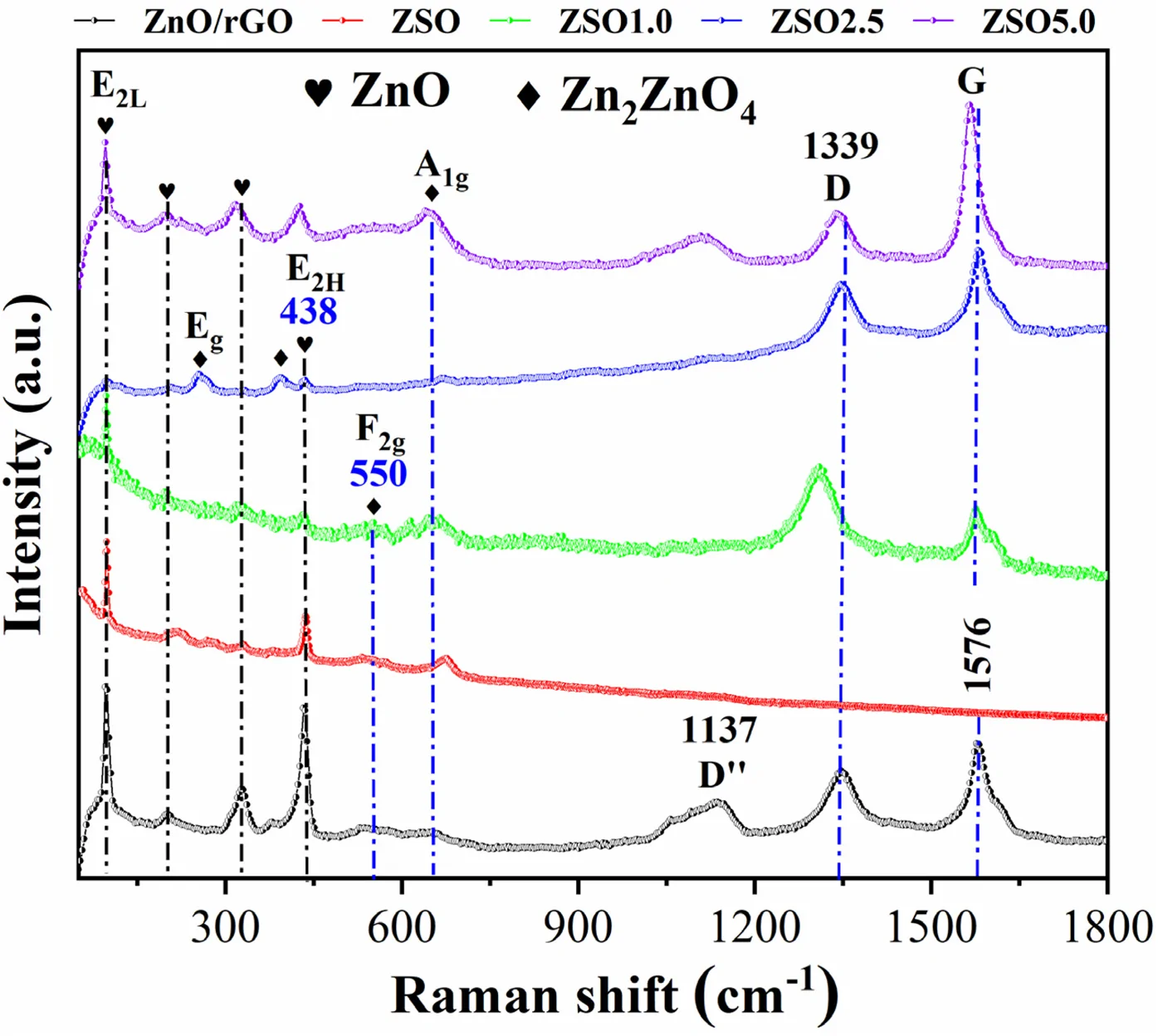
Raman spectra of ZnO/rGO, ZSO, and ZSO/rGO composites with different rGO contents. The labeled peaks correspond to the characteristic modes of ZnO, Zn_2_SnO_4_, and rGO.

For the ZnO/rGO sample, the Raman spectrum is dominated by the characteristic phonon modes of hexagonal wurtzite ZnO. The peaks at approximately 99 and 438 cm^−1^ are assigned to the *E*_2_(low) and *E*_2_(high) modes, respectively.^24,25^ The weak bands at approximately 201 and 324 cm^−1^ are attributed to second-order Raman processes, while the broad feature near 1137 cm^−1^ is associated with higher-order longitudinal-optical-phonon scattering. The pronounced *E*_2_(high) mode is consistent with the formation of crystalline wurtzite ZnO.

The carbon-containing samples exhibit the characteristic D and G bands of rGO at approximately 1339 and 1576 cm^−1^, respectively.^22,24^ The D band is associated with defect-activated breathing vibrations of sp^2^ carbon rings, whereas the G band originates from the in-plane stretching vibration of sp^2^-bonded carbon atoms. The calculated intensity ratio, *I*_D_/*I*_G_ ≈ 0.85, indicates structural disorder and residual defects in the graphene-derived sheets. However, confirmation of GO reduction requires comparison with the *I*_D_/*I*_G_ value of the initial GO or complementary evidence from XPS and FTIR analyses.

For the binary ZSO composite, Raman features associated with both oxide phases are observed. In addition to the ZnO-related modes, bands at approximately 250, 550, and 670 cm^−1^ are assigned to the E_g_, F_2g_, and A_1g_ modes of cubic spinel Zn_2_SnO_4_, respectively.^13,15^ In particular, the high-frequency A_1g_ mode is associated primarily with symmetric stretching vibrations involving the Sn–O framework. These observations support the coexistence of ZnO and Zn_2_SnO_4_, consistent with the XRD results.

For the ternary composites ZSO1.0–ZSO5.0, the characteristic Raman features of ZnO, Zn_2_SnO_4_, and rGO are retained. As the rGO content increases, the D and G bands become more pronounced, while the relative intensities of the oxide-related modes decrease, consistent with the increasing carbon content. No appreciable shifts in the principal ZnO and Zn_2_SnO_4_ Raman modes are observed after rGO incorporation, suggesting that the fundamental crystal structures of the two oxide phases are preserved. ZSO2.5 exhibits identifiable features from the oxide phases and rGO, supporting their coexistence within the ternary composite. The spatial distribution and interfacial contact among the components are further evaluated using electron microscopy and XPS.

### 3.3. FTIR analysis

FTIR spectroscopy was employed to examine the surface functional groups and bonding environments of the prepared samples, as shown in Fig. 3. For pristine ZnO, the absorption band near 512 cm^−1^ is assigned to the Zn–O stretching vibration of hexagonal wurtzite ZnO.^24,25^ The weak feature near 1385 cm^−1^ may originate from residual surface species, whereas the band at approximately 1638 cm^−1^ is associated mainly with the bending vibration of adsorbed water. The broad band in the 3200–3600 cm^−1^ region corresponds to O–H stretching vibrations of surface hydroxyl groups and adsorbed water.

**Fig. 3:**
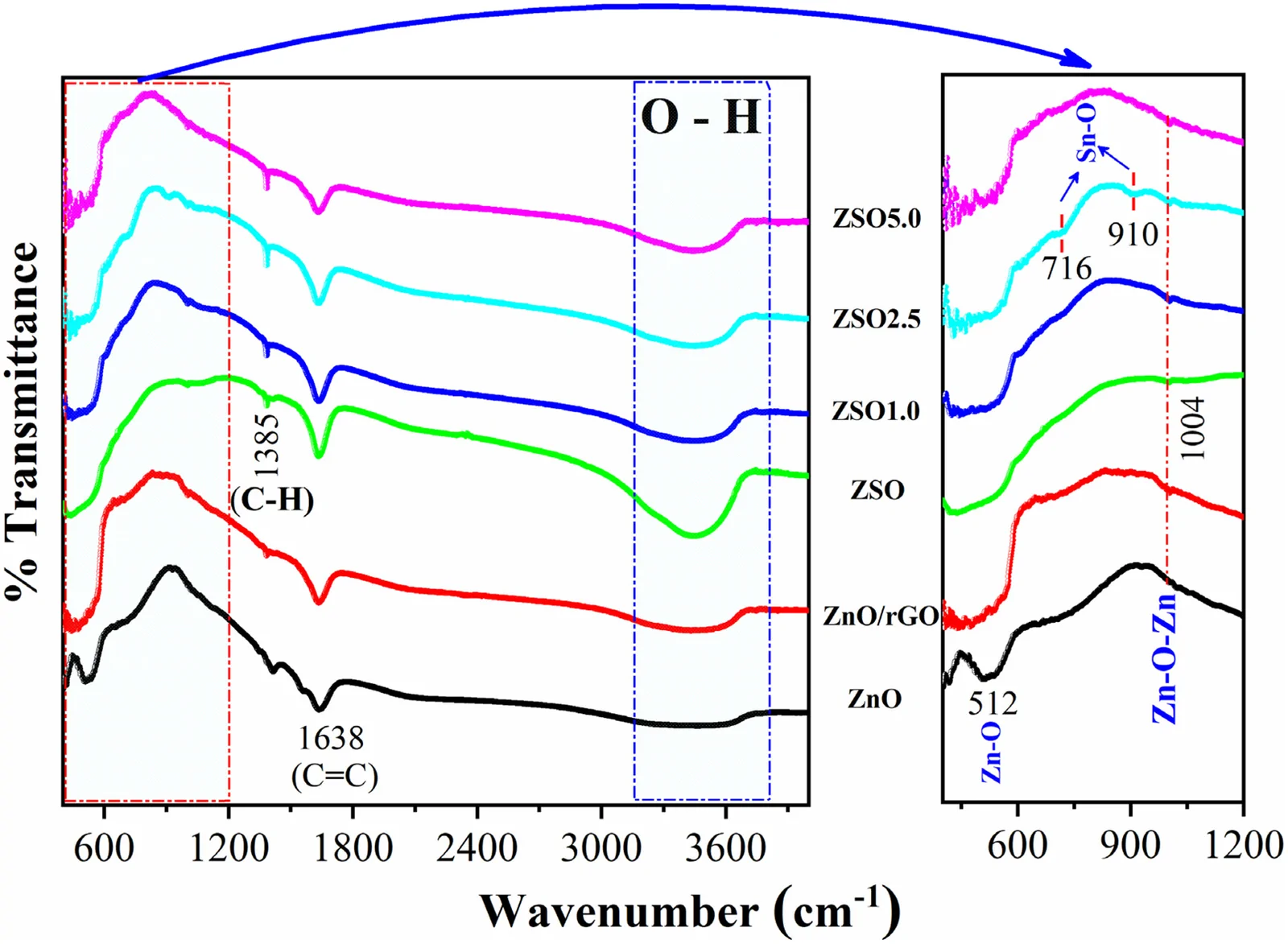
FTIR spectra of ZnO, ZnO/rGO, ZSO, and ZSO/rGO composites with different rGO contents, with an enlarged view of the fingerprint region.

For ZnO/rGO, the band near 1638 cm^−1^ may include a contribution from the skeletal vibration of sp^2^-hybridized C=C domains in rGO, although this feature overlaps with the bending mode of adsorbed water.^22,24^ Therefore, it should be interpreted together with the Raman results rather than regarded as independent evidence of rGO incorporation. The retention of the Zn–O band indicates that the fundamental bonding environment of ZnO is preserved after rGO incorporation.

Compared with pristine ZnO, binary ZSO exhibits changes in the low-wavenumber region, particularly below approximately 800 cm^−1^. These changes reflect differences in the metal–oxygen vibrational environment associated with the coexistence of ZnO and spinel Zn_2_SnO_4_.^11^ The absorption features in this region may contain overlapping contributions from Zn–O, Sn–O, and Zn–O–Sn vibrations, preventing their unambiguous individual assignment.

For the ternary composites ZSO1.0–ZSO5.0, the principal metal–oxygen and surface-related absorption features are retained. The weak bands near 716, 910, and 1004 cm^−1^ may arise from overlapping lattice, surface, and residual oxygen-containing species and should not be assigned exclusively to Zn–O–Zn or Zn–O–Sn vibrations. ZSO2.5 displays contributions from both oxide-related and carbon-related bonding environments. At the highest rGO content, the lower relative intensity of the metal–oxygen bands may be associated with the increased optical contribution or partial surface coverage of the oxide particles by rGO.

Overall, the FTIR results indicate the presence of metal–oxygen bonds, surface hydroxyl groups, adsorbed water, and carbon-related bonding environments. Together with the XRD and Raman results, these observations support the coexistence of ZnO, Zn_2_SnO_4_, and rGO in the ternary composites, although the detailed interfacial bonding configuration cannot be determined from FTIR alone.

### 3.4. FESEM and EDX analyses

The morphology of the ZSO2.5 ternary composite was examined by FESEM, as shown in Fig. 4. Nearly spherical oxide particles with typical diameters of approximately 60–80 nm are distributed over wrinkled, sheet-like structures attributed to rGO, together with a small number of larger aggregates of approximately 200–250 nm. The sheet-like framework may provide anchoring sites for oxide nucleation and restrict extensive particle agglomeration during hydrothermal synthesis.^11,25^

**Fig. 4:**
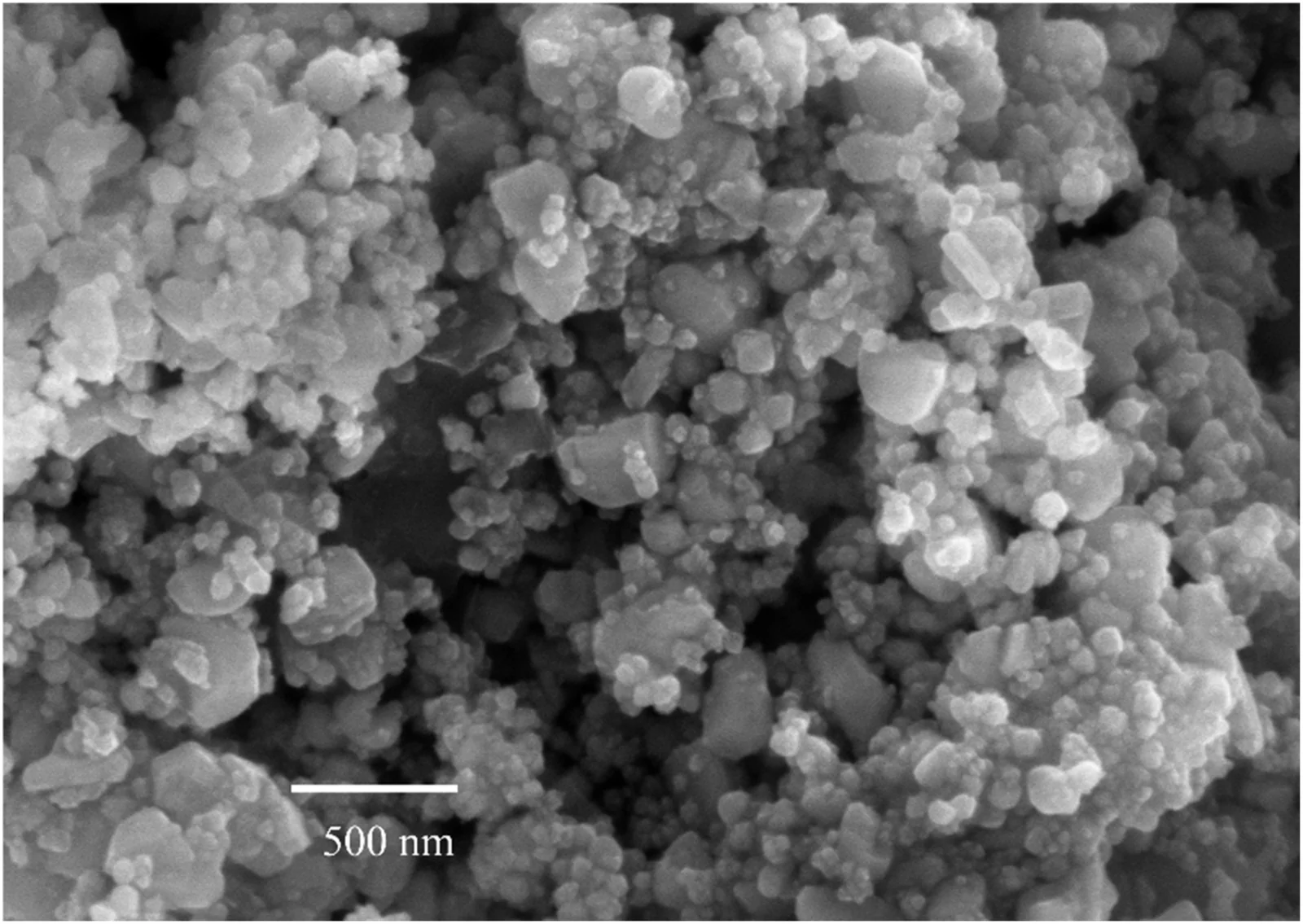
FESEM image of the ZSO2.5 ternary composite showing oxide nanoparticles distributed over wrinkled rGO sheets.

The close contact between the oxide particles and rGO sheets supports the formation of oxide–carbon interfaces. However, the individual ZnO and Zn_2_SnO_4_ particles cannot be distinguished solely from FESEM images. EDX analysis confirms the presence of Zn, Sn, O, and C in the composite, consistent with its expected composition. The observed morphology provides a favorable structural basis for interfacial charge transport, as further evaluated by PL and transient photocurrent measurements.

Energy-dispersive X-ray (EDX) spectroscopy was employed to examine the elemental composition of the synthesized composite, and the corresponding spectrum is presented in Fig. 5. The spectrum shows characteristic signals of C, O, Zn, and Sn, consistent with the expected composition of the ZSO2.5 composite. The C signal is associated primarily with the rGO-containing component, although a possible contribution from the carbon tape or substrate used for EDX analysis cannot be excluded. The Zn, Sn, and O signals are consistent with the oxide phases identified by XRD.

**Fig. 5:**
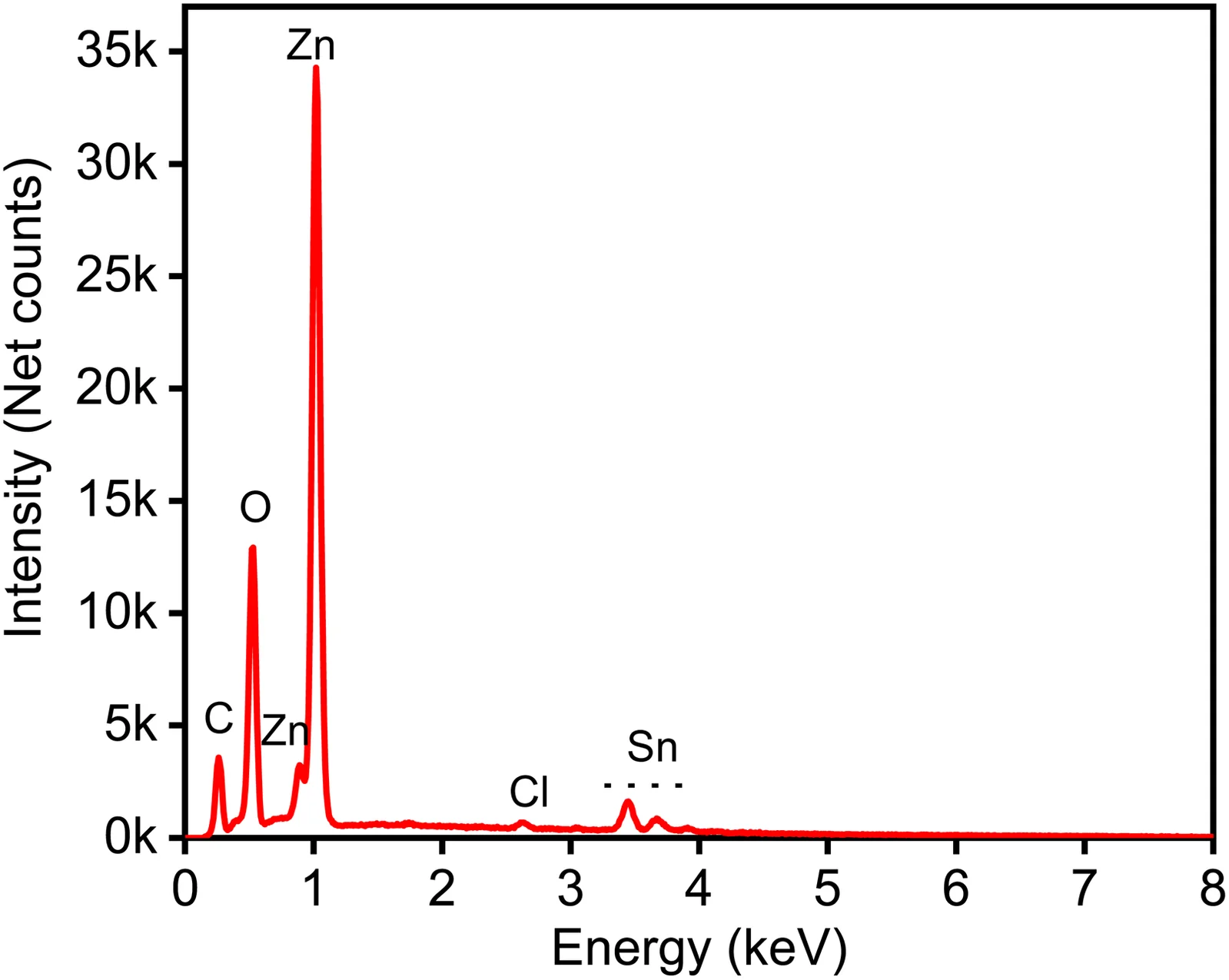
EDX spectrum of the synthesized ZSO2.5 composite confirming the presence of C, O, Zn, and Sn.

A weak Cl signal with a concentration of approximately 0.7 at% is also detected and may originate from residual chloride-containing precursor species. Because of its low concentration, Cl is not detected as a separate crystalline phase by XRD. However, its possible influence on the material properties cannot be evaluated from EDX alone.

The semi-quantitative EDX results are summarized in Table 3. The measured atomic concentrations of C, O, Zn, and Sn are 15.0, 45.7, 34.4, and 4.2 at%, respectively, corresponding to 4.9, 19.9, 61.1, and 13.4 wt%. The resulting Zn/Sn atomic ratio is approximately 8.2, indicating that the analyzed region is Zn-rich relative to stoichiometric Zn_2_SnO_4_. This observation is qualitatively consistent with the coexistence of excess ZnO and Zn_2_SnO_4_ identified by XRD. Nevertheless, because EDX provides local and semi-quantitative compositional information, the measured ratio should not be interpreted as the exact bulk stoichiometry of the composite.

**Table 3:** Semi-quantitative elemental composition of the ZSO2.5 ternary composite determined by EDX analysis

Element	X-ray line	Atomic content (at%)	Weight content (wt%)	Net counts	Error (at%)	Error (wt%)
C	K	15.0	4.9	21 205	0.2	0.1
O	K	45.7	19.9	81 196	0.3	0.1
Cl	K	0.7	0.7	3190	0.0	0.0
Zn	L	34.4	61.1	283 655	0.1	0.3
Sn	L	4.2	13.4	23 595	0.1	0.3
**Total**	—	**100.0**	**100.0**	—	—	—

Overall, the complementary FESEM, EDX, and XRD results support the coexistence of the expected morphological, elemental, and crystalline features of the ZSO2.5 composite. Their relationship with charge-carrier separation and photocatalytic performance is further evaluated using PL, transient photocurrent, and photocatalytic measurements.

### 3.5. TEM analysis

Fig. 6 presents TEM images of pristine ZnO and the ZSO2.5 ternary composite. As shown in Fig. 6(a), pristine ZnO consists of irregular polygonal and short rod-like particles with sizes of approximately 100–250 nm. The particles exhibit distinct boundaries and appreciable agglomeration, which may be associated with their high surface energy.

**Fig. 6:**
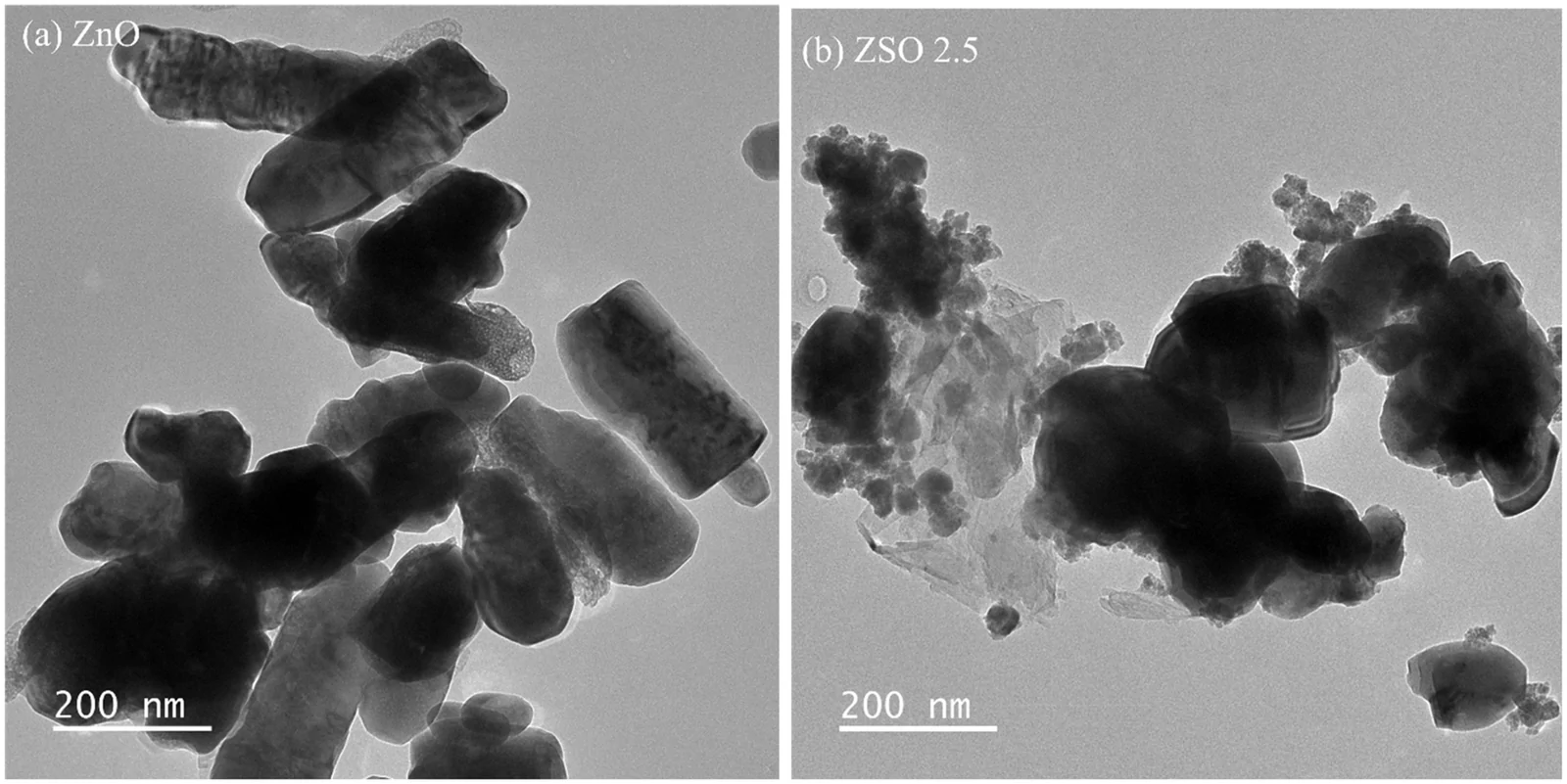
TEM images of (a) pristine ZnO and (b) the ZSO2.5 ternary composite.

In contrast, ZSO2.5 (Fig. 6(b)) exhibits oxide particles distributed over thin, transparent, and wrinkled sheet-like structures attributed to rGO. This morphology, together with the Raman results, supports the successful incorporation of rGO into the composite. Compared with pristine ZnO, the oxide particles appear less aggregated and more dispersed in the presence of rGO, suggesting that the graphene-derived sheets restrict particle coalescence and provide anchoring surfaces during hydrothermal growth.^24–26^

The close contact between the oxide particles and rGO sheets provides a structural basis for the formation of oxide–carbon interfaces. However, conventional TEM images alone cannot distinguish ZnO from Zn_2_SnO_4_ or directly demonstrate charge transfer. The possible contribution of these interfaces to charge separation and transport is therefore evaluated using the PL and transient photocurrent results presented in the subsequent sections.

### 3.6. X-ray photoelectron spectroscopy analysis

XPS measurements, including survey and high-resolution core-level spectra, were performed on pristine ZnO and ZSO2.5, representing the reference sample and the composite exhibiting the highest photocatalytic activity, respectively. These measurements provide information about surface elemental composition, chemical states, and possible interfacial electronic interactions. As shown in Fig. 7, Zn and O signals are detected in pristine ZnO, whereas additional Sn and C signals are observed for ZSO2.5. Together with the XRD and Raman results, these signals support the presence of Zn_2_SnO_4_ and rGO in the ternary composite.

**Fig. 7:**
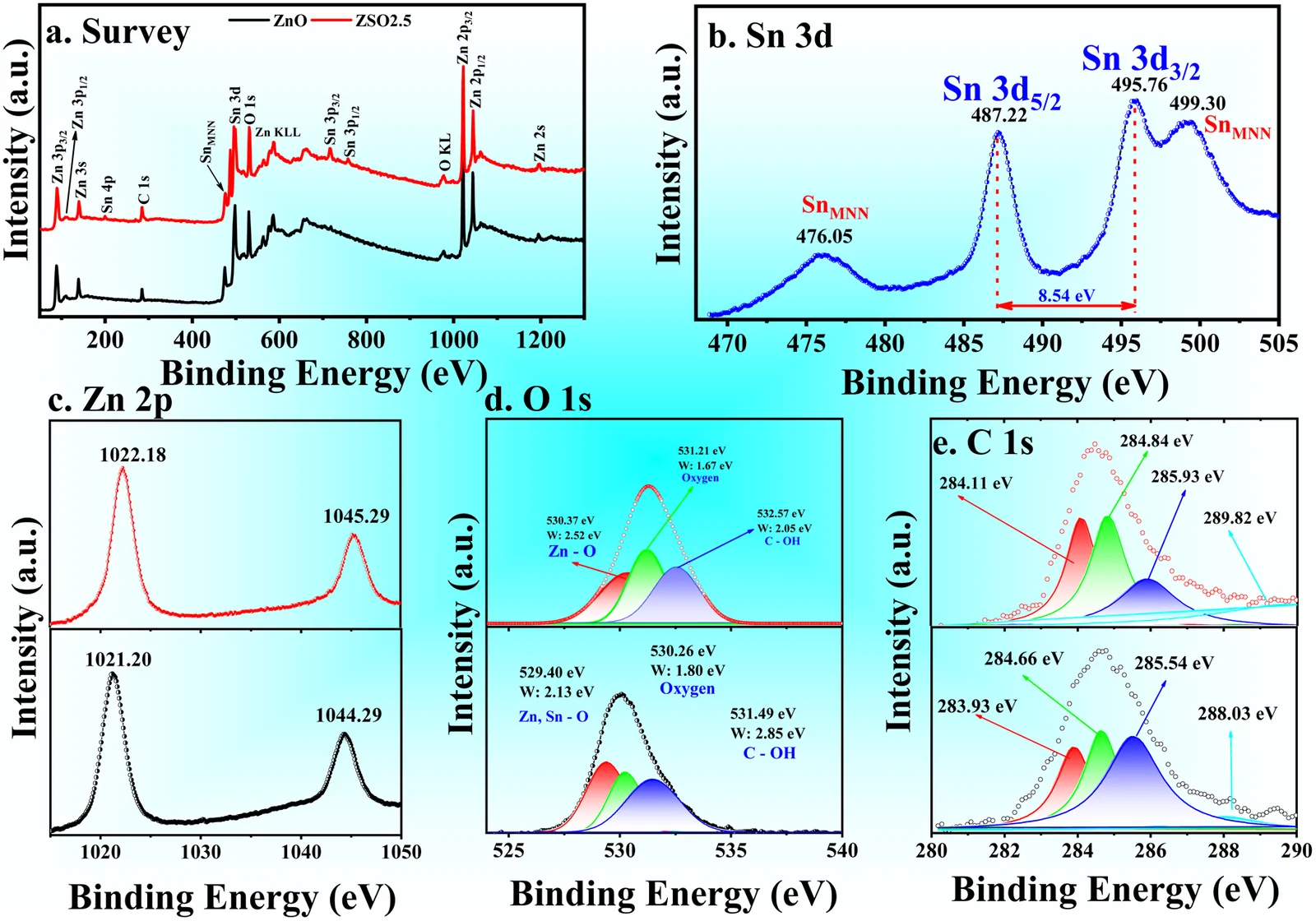
XPS spectra of pristine ZnO and ZSO2.5: (a) survey spectra and high-resolution spectra of (b) Zn 2p, (c) Sn 3d, (d) O 1s, and (e) C 1s.

#### 3.6.1. Sn 3d and Zn 2p core levels

The high-resolution Sn 3d spectrum of ZSO2.5 exhibits two peaks centered at 487.22 and 495.76 eV, assigned to Sn 3d_5/2_ and Sn 3d_3/2_, respectively, with a spin–orbit separation of approximately 8.54 eV. These features are consistent with Sn^4+^ species in an oxide environment.^27,28^ No distinct components attributable to Sn^2+^ or metallic Sn are resolved within the sensitivity and fitting resolution of the measurement. Combined with the XRD results, the Sn^4+^ state supports the presence of the Zn_2_SnO_4_ phase.

The Zn 2p spectrum of pristine ZnO shows peaks at 1021.20 and 1044.29 eV, assigned to Zn 2p_3/2_ and Zn 2p_1/2_, respectively, and characteristic of Zn^2+^ in an oxide environment.^25,29^ For ZSO2.5, the corresponding peaks are located at 1022.18 and 1045.29 eV. Provided that both spectra were calibrated using the same reference and measured under comparable charging conditions, this positive shift suggests a change in the local electronic environment of Zn after Zn_2_SnO_4_ and rGO incorporation. However, the binding-energy shift alone does not establish the direction or magnitude of interfacial charge transfer.

#### 3.6.2. O 1s and C 1s spectra

The O 1s spectrum of pristine ZnO is deconvoluted into components at approximately 530.3, 531.2, and 532.6 eV. The low-binding-energy component is assigned to lattice oxygen in the metal–oxide framework. The component near 531.2 eV is attributed cautiously to defect-related or surface-active oxygen species, whereas the higher-binding-energy component may arise from surface hydroxyl groups, adsorbed water, and other weakly bound oxygen-containing species.^22,25,29^ A similar multicomponent profile is observed for ZSO2.5. Because several surface species contribute within overlapping binding-energy ranges, the O 1s spectrum alone cannot provide definitive identification or quantitative determination of oxygen vacancies.

The C 1s spectrum of ZSO2.5 is deconvoluted into components centered at 284.84, 285.93, and 289.82 eV, assigned to C–C/C=C, C–O, and highly oxidized carbon species such as O–C=O, respectively.^22,25^ The dominant C–C/C=C component is consistent with a conjugated rGO framework, whereas the remaining oxygen-containing groups may provide anchoring sites for oxide particles. Nevertheless, confirmation of GO reduction requires comparison with the C 1s component fractions or C/O ratio of the initial GO. The weak C 1s signal in pristine ZnO is attributed mainly to adventitious surface carbon.

#### 3.6.3. Evidence of interfacial electronic interaction

The XPS results indicate changes in the surface chemical environment after combining ZnO with Zn_2_SnO_4_ and rGO. In particular, the Zn 2p binding-energy shift, if confirmed after consistent charge correction, is compatible with interfacial electronic redistribution in ZSO2.5. The Sn^4+^ signal, sp^2^-dominated carbon component, and multicomponent O 1s profile further support the coexistence of the oxide and carbonaceous components.

XPS alone does not establish the complete charge-transfer pathway or directly demonstrate suppressed electron–hole recombination. Therefore, the possible interfacial electronic interaction inferred from XPS is evaluated together with the PL and transient photocurrent results. Collectively, these complementary observations support improved charge separation and interfacial carrier transport in ZSO2.5.

### 3.7. Optical absorption and apparent transition-energy analysis

The optical absorption characteristics of the ZSO and ZSO/rGO composites were investigated using UV-vis spectroscopy. As shown in Fig. 8(a), all samples exhibit strong absorption in the ultraviolet region, with spectral features near 269 and 380 nm. The absorption edge near 380 nm is associated primarily with the near-band-edge transition of ZnO.^24,25^ The higher-energy feature near 269 nm may contain contributions from electronic transitions involving the Zn_2_SnO_4_ phase. However, because 269 nm corresponds to an energy of approximately 4.61 eV, this feature should not be directly assigned as the Zn_2_SnO_4_ band edge without additional phase-resolved optical evidence.^11,27,30^

**Fig. 8:**
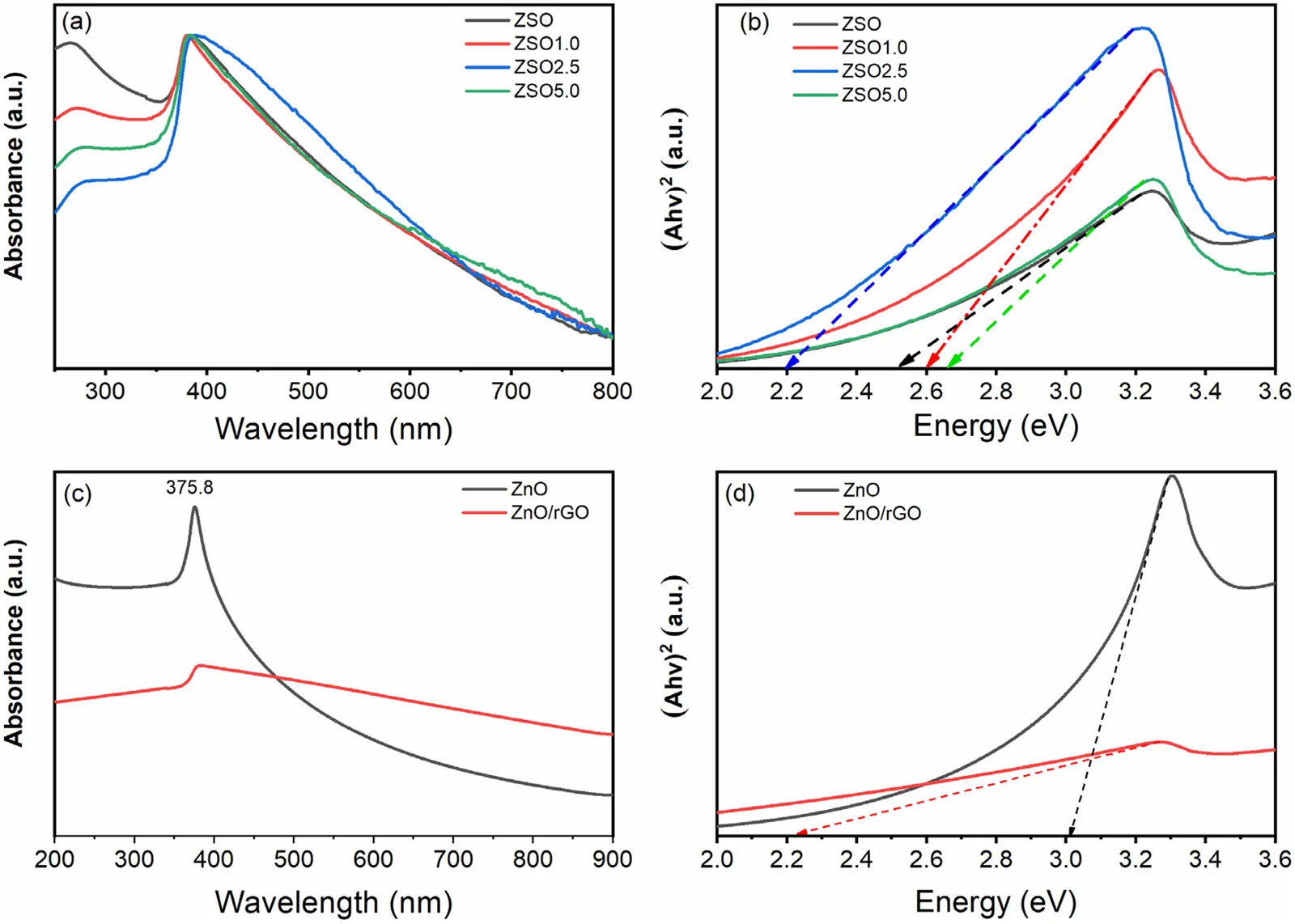
(a) UV-vis absorbance spectra and (b) corresponding (*Ahν*)^2^*versus* photon energy (*hν*) plots for the binary ZSO and ternary ZSO/rGO composites with different rGO loadings; (c) UV-vis absorbance spectra and (d) corresponding (*Ahν*)^2^*versus* photon energy (*hν*) plots for pristine ZnO and ZnO/rGO.

The relative intensity ratio *I*_269_/*I*_380_ varies non-monotonically with rGO content. UV-vis intensity can be affected by particle concentration, dispersion, agglomeration, and light scattering. Therefore, this variation alone does not demonstrate that rGO promotes ZnO crystallization or suppresses Zn_2_SnO_4_ growth; such an interpretation should instead be based on the XRD and microscopic results.

The rGO-containing composites also exhibit changes in the visible-region absorption background, which may be associated with the broadband absorption of the π-conjugated carbon network and its interaction with the oxide components.^22,24,25^ Because both ZnO and Zn_2_SnO_4_ are wide-band-gap semiconductors, this response should not be interpreted as intrinsic visible-light band-to-band absorption of the oxide phases. At 5.0 wt% rGO, the lower measured optical response may result from particle aggregation, surface coverage, light shielding, or differences in sample dispersion. This behavior is therefore interpreted cautiously and considered together with the structural and photocatalytic results.

The apparent optical transition energies were estimated using a Tauc-type analysis based on the measured absorbance (*A*). For a direct allowed transition, the relationship was expressed as:(*Ahν*)^2^ = *B*(*hν* − *E*_g_)where *A* is the measured absorbance, *hν* is the photon energy, *B* is a proportionality constant, and *E*_g_ is the apparent optical transition energy. The apparent *E*_g_ values were estimated by extrapolating the selected linear region of the (*Ahν*)^2^*versus hν* plots to the photon-energy axis. Because absorbance rather than the intrinsic absorption coefficient was used, the resulting values are discussed as apparent optical transition energies rather than intrinsic band-gap energies.

The corresponding (*Ahν*)^2^*versus hν* plots for the ZSO and ZSO/rGO composites are shown in Fig. 8(b), and the estimated apparent optical transition energies are summarized in Table 4. Binary ZSO exhibits an apparent transition energy of 2.52 eV. After the incorporation of 1.0 wt% rGO, this value increases slightly to 2.61 eV, whereas ZSO2.5 exhibits a lower value of 2.20 eV, representing a decrease of approximately 0.32 eV relative to ZSO. When the rGO content is increased to 5.0 wt%, the apparent transition energy increases to 2.67 eV. This non-monotonic variation indicates that the Tauc response is influenced by rGO content. Possible contributing factors include changes in the optical absorption background, defect-related transitions, light scattering, particle aggregation, and surface coverage by rGO. ZSO2.5 exhibits both the lowest apparent transition energy and the highest MB removal efficiency; however, this correlation does not establish a direct causal relationship.

**Table 4:** Apparent optical transition energies of ZnO, ZnO/rGO, ZSO, and ZSO/rGO composites estimated from the absorbance-based Tauc-type analysis

Sample	Apparent optical transition energy (eV)
ZnO	3.02
ZnO/rGO	2.23
ZSO	2.52
ZSO1.0	2.61
ZSO2.5	2.20
ZSO5.0	2.67

These values should be regarded as apparent optical transition energies of the composites rather than the intrinsic band gaps of the individual ZnO and Zn_2_SnO_4_ phases. Both ZnO and Zn_2_SnO_4_ are wide-band-gap semiconductors that absorb primarily in the ultraviolet region.^11,27,30,31^ Therefore, the visible-light response of the ternary composites cannot be explained solely by intrinsic band-gap narrowing. The broadband absorption of rGO, defect-related transitions, and interfacial electronic interactions may influence the measured optical response, while carrier transport may contribute to the photocatalytic behavior.^18,22,24,25^ Possible MB photosensitization under visible-light irradiation also cannot be excluded. Because VB-XPS, UPS, and Mott–Schottky measurements were not performed, the band-edge positions and interfacial charge-transfer pathway cannot be conclusively determined.

Fig. 8(c) presents the UV-vis absorption spectra of pristine ZnO and ZnO/rGO. Pristine ZnO exhibits a distinct absorption edge at approximately 375.8 nm, associated with the near-band-edge transition of wurtzite ZnO.^24,25,31^ Beyond this edge, the absorption decreases rapidly, indicating its limited intrinsic visible-light-harvesting capability.

After rGO incorporation, the ZnO absorption edge becomes less distinct, while ZnO/rGO exhibits a broader absorption background across the visible region from 400 to 900 nm. This behavior is attributed primarily to the broadband absorption of the π-conjugated rGO network and its interaction with ZnO.^18,22,24,25^ Defect-related and interfacial states may also influence the absorption profile. Thus, rGO should be regarded mainly as a broadband light-absorbing and conductive component rather than as a species that intrinsically modifies the crystalline band structure of ZnO.

For pristine ZnO and ZnO/rGO, the corresponding (*Ahν*)^2^*versus hν* plots are shown in Fig. 8(d). Extrapolation of the selected linear regions gives apparent transition energies of approximately 3.02 eV for pristine ZnO and 2.23 eV for ZnO/rGO. The lower value obtained for the composite should not be interpreted as evidence that the intrinsic ZnO band gap decreases by 0.79 eV. Instead, it reflects the combined optical response of ZnO and rGO, including broadband rGO absorption, defect-related states, interfacial contributions, and uncertainty in selecting the linear Tauc region.^22,24,25^ Similarly, the difference between the ZnO absorption onset near 375.8 nm and its Tauc-derived value of 3.02 eV may arise from absorption-tail contributions and the extrapolation procedure. The intrinsic band gap of crystalline ZnO is therefore expected to remain close to its typical value of approximately 3.2–3.3 eV.

Although ZnO and Zn_2_SnO_4_ are wide-band-gap semiconductors that intrinsically absorb mainly in the ultraviolet region, the rGO-containing composites exhibit a substantially broader absorption background in the visible region, as demonstrated by the comparison between ZnO and ZnO/rGO in Fig. 8(c). Therefore, the visible-light-assisted activity of ZSO2.5 should not be attributed to intrinsic band-gap narrowing of ZnO or Zn_2_SnO_4_. Instead, it likely arises from the combined contributions of the broadband optical absorption of the π-conjugated rGO network, defect- and interface-related electronic states, enhanced interfacial carrier transport, and possible MB photosensitization. The apparent transition energies obtained from the Tauc plots consequently represent the overall optical response of the composites rather than the intrinsic band gaps of the individual oxide phases. Because VB-XPS, UPS, and Mott–Schottky measurements were not performed, the exact band-edge positions and interfacial charge-transfer direction cannot be conclusively determined in the present study.

### 3.8. Photoluminescence spectroscopy analysis

Steady-state photoluminescence spectroscopy was employed to investigate the radiative recombination behavior and defect-related emissions of the prepared samples. As shown in Fig. 9(a) and (b), the spectra exhibit two principal emission features associated mainly with ZnO: a near-band-edge emission at approximately 380 nm and a broad deep-level emission extending from approximately 450 to 720 nm, with a maximum near 550 nm. The near-band-edge emission is attributed to excitonic or band-edge recombination, whereas the broad deep-level band is generally associated with native defects and surface-related states in ZnO.^25,29,31^ Because several defect states contribute within overlapping spectral ranges, this broad band cannot be assigned uniquely to individual defects based on steady-state PL alone.

**Fig. 9:**
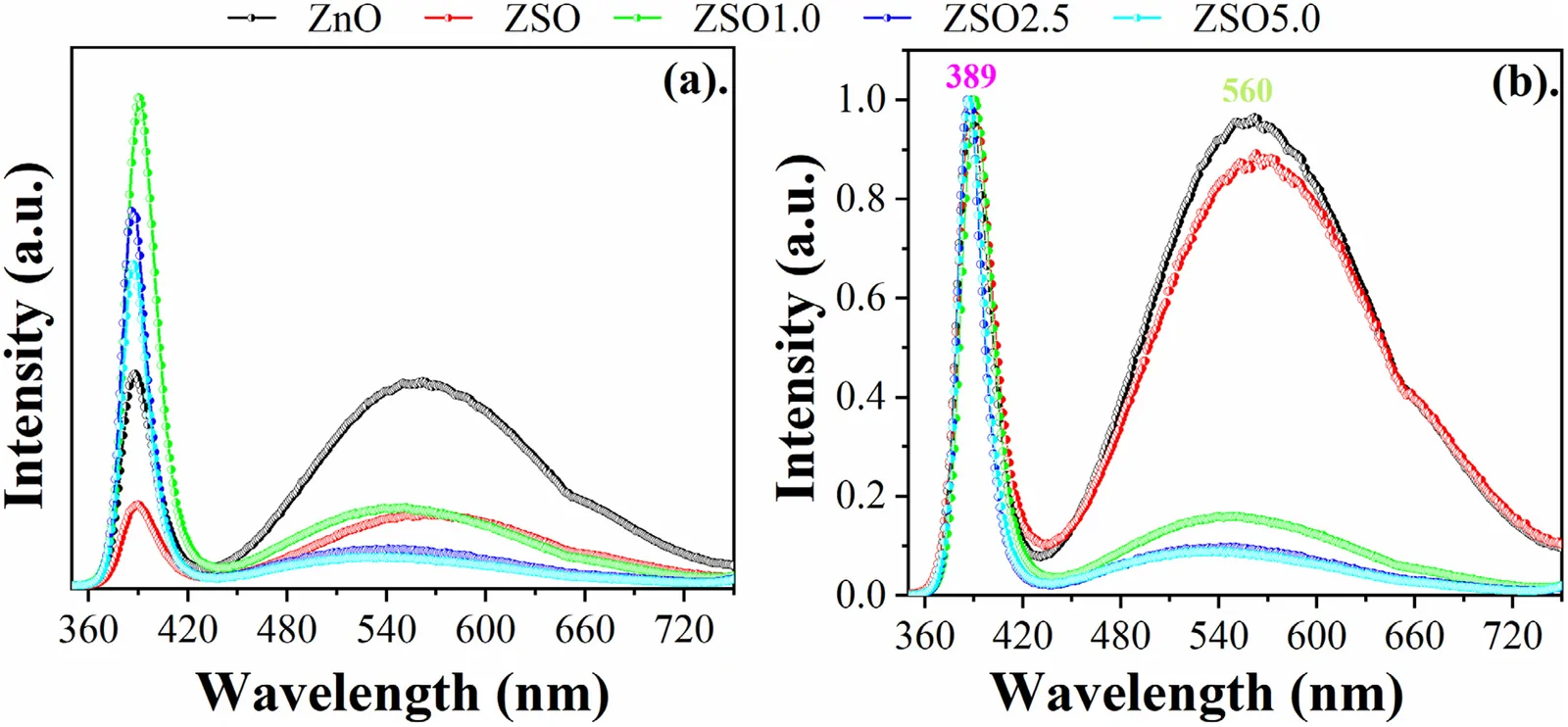
(a) Steady-state and (b) normalized PL spectra of ZnO, ZSO, and ZSO/rGO composites with different rGO contents. The normalized spectra highlight changes in spectral shape and peak position.

Under identical measurement conditions, pristine ZnO exhibits relatively intense near-band-edge and deep-level emissions, indicating appreciable band-edge and defect-mediated radiative recombination. After the formation of binary ZSO, the PL intensity decreases markedly, while the near-band-edge peak position remains nearly unchanged. This suppression is consistent with altered carrier-recombination pathways following the formation of the oxide–oxide interface.^11,27,32^ The nearly unchanged peak position also suggests that the PL quenching is related more closely to changes in recombination behavior than to intrinsic modification of the ZnO band gap. Nevertheless, steady-state PL alone cannot determine the band alignment or direction of interfacial charge transfer.

The ternary ZSO/rGO composites exhibit further PL quenching, with ZSO2.5 showing the lowest emission intensity. The conductive π-conjugated rGO network may provide interfacial pathways for carrier extraction and transport.^22,24,25^ However, rGO can also influence measured PL intensity through optical absorption, energy transfer, and nonradiative trapping. Therefore, PL quenching alone should not be regarded as conclusive evidence of improved charge separation.

A slight shift in the deep-level emission maximum is observed after rGO incorporation, suggesting a change in the relative contributions of defect- and surface-related states. This shift should be interpreted cautiously because of the broad and overlapping nature of the emission band. When considered together with the enhanced transient photocurrent response, the PL quenching supports improved carrier separation and transport in ZSO2.5. Direct time-resolved measurements would nevertheless be required to determine carrier lifetimes and transfer kinetics quantitatively.

### 3.9. Transient photocurrent response and charge-transport behavior

Transient photocurrent measurements were performed over repeated light-on/light-off cycles to evaluate the photoinduced charge response. As shown in Fig. 10, both binary ZSO and ZSO2.5 exhibit rapid and reproducible switching responses, with the photocurrent returning close to its baseline after illumination is switched off. This behavior indicates stable short-term photoresponse under the investigated conditions.

**Fig. 10:**
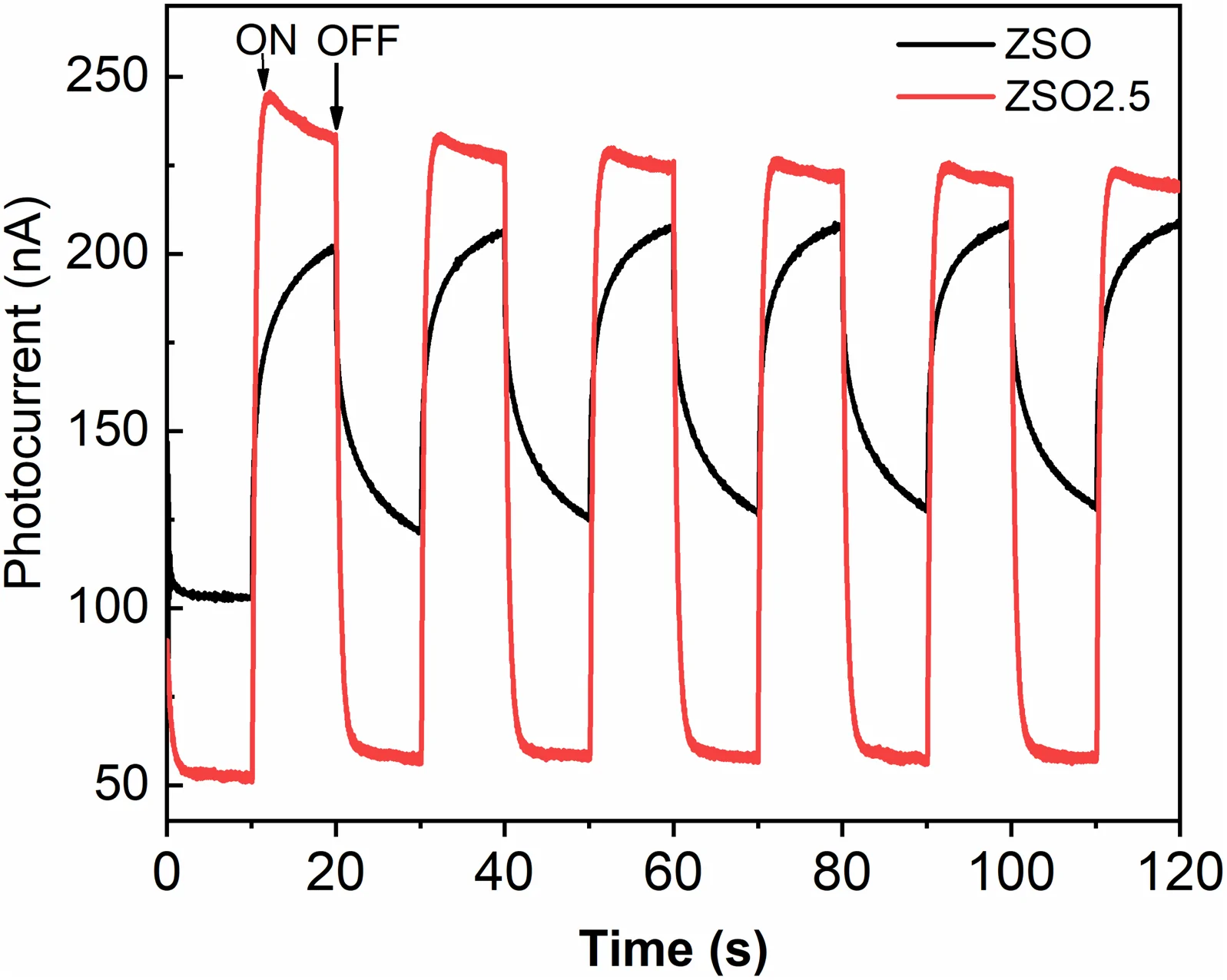
Transient photocurrent responses of ZSO and ZSO2.5.

Under identical measurement conditions, ZSO2.5 exhibits a markedly higher photocurrent response than binary ZSO throughout all irradiation cycles. This enhancement is consistent with improved overall generation, separation, transport, and collection of photoinduced charge carriers after rGO incorporation.^18,25,29^ In the ternary ZSO/rGO composite, rGO may act as a conductive charge-transport mediator, providing interfacial pathways for carrier migration and reducing recombination losses.^17,22,25,33^

Although the ZnO/Zn_2_SnO_4_ interface may promote charge separation, binary ZSO lacks the additional conductive pathways provided by rGO. Its lower photocurrent therefore indicates less effective charge extraction and collection under the same conditions. However, photocurrent magnitude can also be influenced by electrode loading, film thickness, active area, light absorption, and electrode–electrolyte contact. These parameters must therefore be maintained consistently when comparing the samples.

The reproducible response of ZSO2.5 over successive cycles indicates good short-term photoresponse stability. When considered together with the PL quenching, the enhanced photocurrent supports improved carrier separation and transport in the ternary composite. Nevertheless, transient photocurrent measurements alone cannot determine the direction of interfacial charge transfer or conclusively distinguish between Type-II and Z-scheme-like mechanisms.

### 3.10. Photocatalytic MB removal, kinetics, and reusability

The photocatalytic performance of the prepared samples was evaluated through the removal and decolorization of methylene blue (MB) under visible-light irradiation. Before illumination, each photocatalyst/MB suspension was stirred in the dark for 60 min to establish adsorption–desorption equilibrium. Fig. 11 presents the time-dependent UV-vis absorption spectra of MB using the optimized ZSO2.5 photocatalyst as a representative example; the corresponding spectra for the other samples are provided in Fig. S1 of the SI. The characteristic MB absorption bands at approximately 664 and 612 nm gradually decrease with irradiation time, indicating progressive decolorization and removal.^11,22,34^ The absorbance values at 664 nm were used to calculate the removal efficiencies and apparent kinetics presented in Fig. 12, with C_0_ defined as the MB concentration after dark equilibration.

**Fig. 11:**
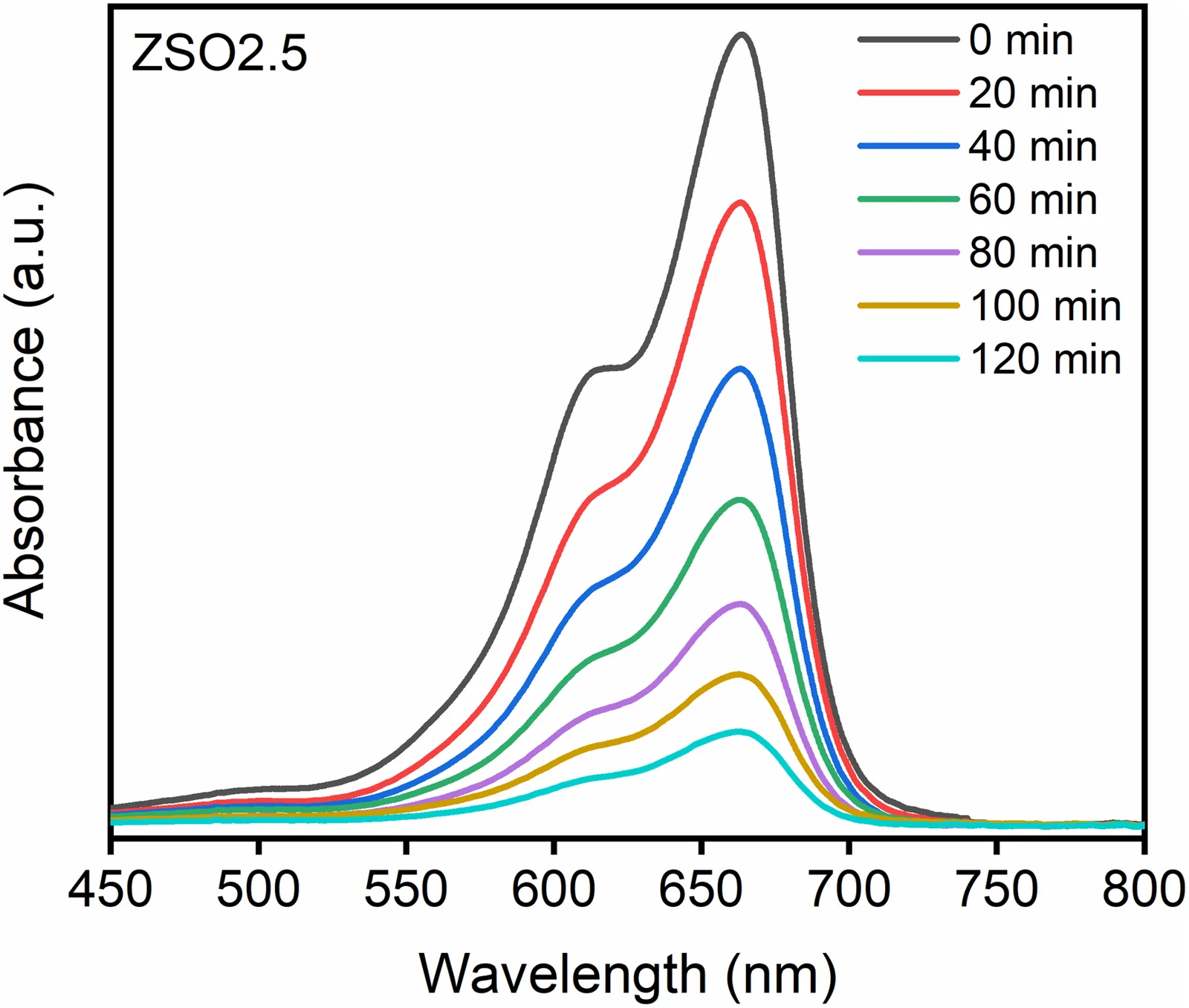
Time-dependent UV-vis absorption spectra of MB during visible-light-assisted removal using the ZSO2.5 photocatalyst.

**Fig. 12:**
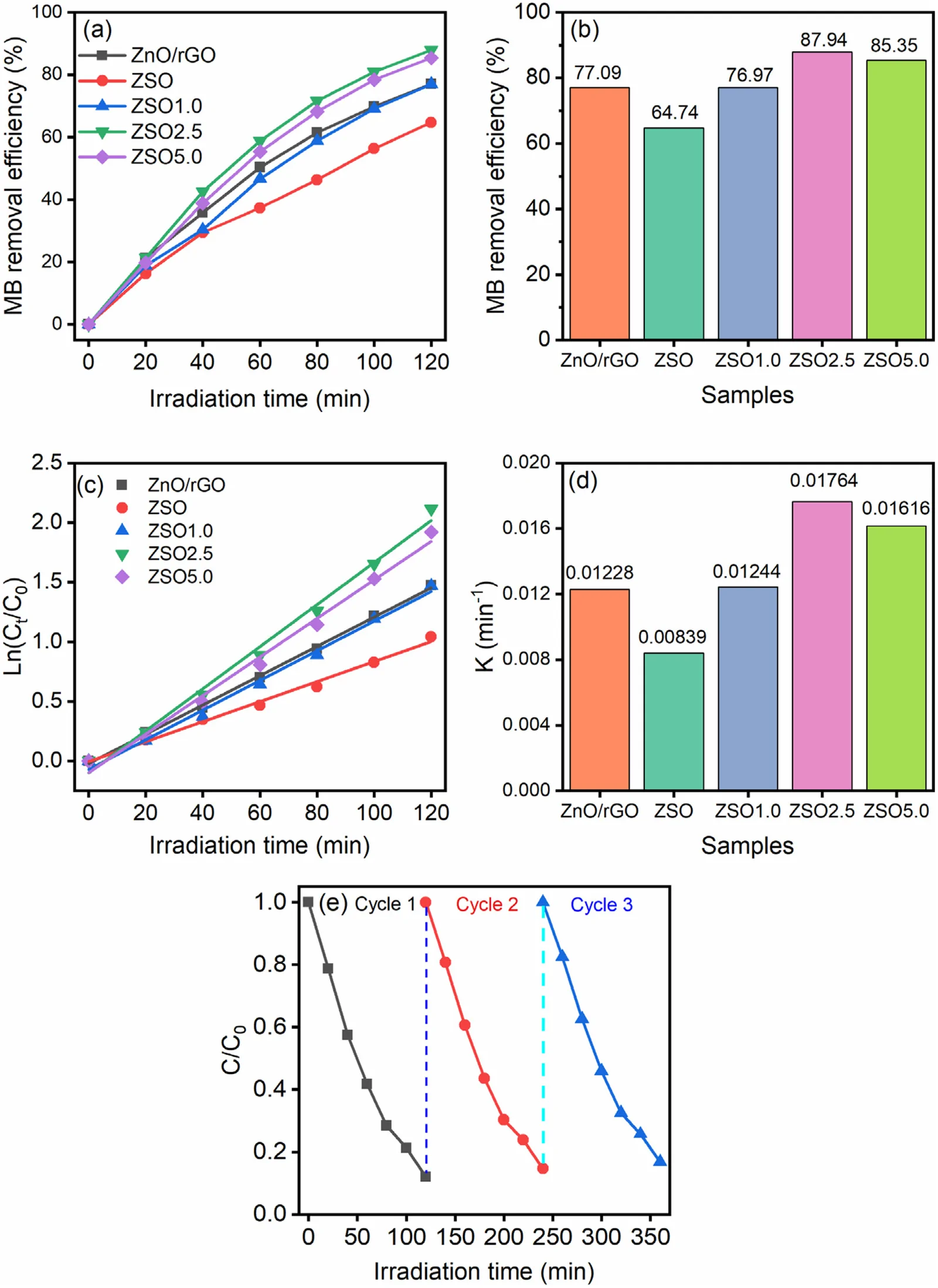
(a) Time-dependent MB removal profiles; (b) removal efficiencies; (c) apparent pseudo-first-order kinetic plots; (d) apparent rate constants (*k*_app_); and (e) reusability of ZSO2.5 over three consecutive cycles under visible-light irradiation.

The decrease in MB absorbance demonstrates the loss of its characteristic chromophoric absorption but does not identify the transformation products or confirm complete degradation or mineralization. Similar limitations have been highlighted in recent reviews of spinel-based photocatalysts for MB removal, in which adsorption, photocatalytic transformation, operating conditions, and mineralization are recognized as distinct aspects of the overall removal process.^14^ HPLC or LC-MS analyses would be required to identify the transformation products and clarify the molecular transformation pathway, while total organic carbon analysis would be necessary to determine the mineralization degree. These measurements were beyond the scope of the present study.

As shown in Fig. 12, the apparent kinetics and removal efficiencies depend strongly on catalyst composition. ZSO2.5, containing 2.5 wt% rGO, exhibits the highest performance, achieving an MB removal efficiency of 87.94% after 120 min and an apparent rate constant of *k*_app_ = 0.01764 min^−1^. The approximately linear relationship between ln(*C*_0_/*C*_*t*_) and irradiation time indicates that the data can be described by an apparent pseudo-first-order kinetic model under the investigated experimental conditions. Here, *C*_*t*_ denotes the MB concentration at irradiation time *t*, estimated from the absorbance at 664 nm. Because the adsorption equilibrium and intrinsic surface-reaction constants were not determined independently, this kinetic fit does not constitute definitive confirmation of a Langmuir–Hinshelwood mechanism.

The results indicate an optimum rGO content. At 1.0 wt% rGO, the available oxide–rGO interfacial area and conductive pathways may be insufficient to maximize carrier transport. Conversely, increasing the rGO content to 5.0 wt% may promote surface coverage, aggregation, and light shielding, thereby reducing photon utilization and active-site accessibility.^11,22,25,35–37^ These explanations represent possible mechanisms and should be considered together with the structural, optical, PL, and photocurrent results.

Among the selected reference and optimized samples, the activity follows the order ZSO2.5 > ZnO/rGO > ZSO. The higher performance of ZSO2.5 suggests a combined contribution from the ZnO/Zn_2_SnO_4_ interface and the conductive rGO network. The graphene-derived sheets may facilitate carrier transport and provide additional MB adsorption sites through π–π interactions with their sp^2^ carbon framework.^17,22,25^ This interpretation is supported by the lower PL intensity and higher transient photocurrent response of ZSO2.5. However, the available measurements do not establish the band-edge positions or direction of interfacial carrier migration.

The reusability of ZSO2.5 was evaluated over three consecutive cycles, as shown in Fig. 12(e). After each cycle, the photocatalyst was recovered by centrifugation, washed with deionized water and ethanol, dried at 80 °C, and reused under identical conditions. Only a limited change in removal efficiency and apparent kinetics is observed, indicating good short-term operational stability.

Because post-cycling structural and surface analyses were not performed, preservation of the crystal structure, retention of rGO, and suppression of ZnO photocorrosion cannot be confirmed. Longer cycling tests, catalyst-mass recovery measurements, and post-reaction characterization are required to establish long-term durability.^17,20,31^ Thus, the three-cycle experiment demonstrates short-term reusability but is insufficient to confirm long-term stability under practical water-treatment conditions.

### 3.11. Reactive-species trapping and proposed photocatalytic mechanism

#### 3.11.1. Reactive-species trapping analysis

Scavenger experiments were conducted to evaluate the involvement of reactive species in the visible-light-assisted removal of MB over ZSO2.5. As shown in Fig. 13(a), the MB removal efficiency reached 87.94% after 120 min without scavengers. After the addition of benzoquinone, isopropanol, and EDTA, the efficiency decreased to 42.36%, 52.65%, and 78.69%, respectively.

**Fig. 13:**
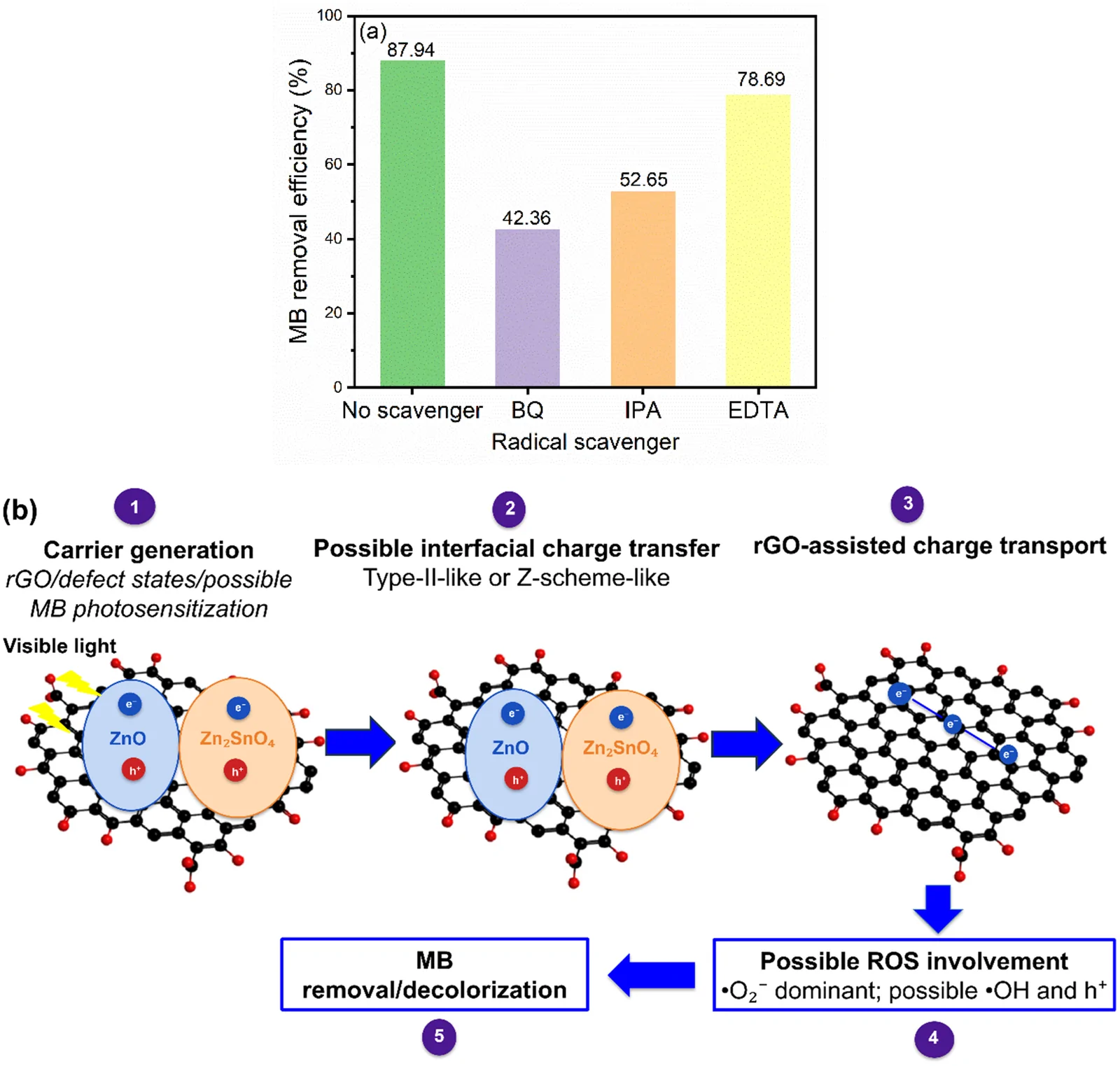
(a) Reactive-species trapping experiments for MB removal over ZSO2.5 using BQ, IPA, and EDTA; (b) possible Type-II-like or Z-scheme-like interfacial charge-transfer pathways and the proposed visible-light-assisted MB removal process over the ZSO/rGO heterostructure. The illustrated pathways represent plausible models based on the available experimental results and literature data rather than a definitive mechanistic assignment.

Benzoquinone produced the strongest inhibition, followed by isopropanol and EDTA, suggesting the qualitative importance order ˙O_2_^−^ > ˙OH > h^+^ under the investigated conditions. Thus, ˙O_2_^−^ appears to make the most substantial contribution, while ˙OH and h^+^ may also participate. However, scavengers differ in selectivity, reaction kinetics, adsorption behavior, and effects on the catalyst surface. These results therefore provide qualitative evidence of reactive-species involvement rather than quantitative contributions to the overall process.^15,38^

#### 3.11.2. Possible interfacial charge-transfer pathways

Fig. 13(b) illustrates possible interfacial charge-transfer pathways in the ZSO/rGO composite. The oxide particles are in close contact with the rGO sheets, forming oxide–oxide and oxide–carbon interfaces that may facilitate interfacial carrier separation and transport.^11,27,28,32,39^ Because ZnO and Zn_2_SnO_4_ are wide-bandgap semiconductors, their direct band-to-band excitation under visible light is expected to be limited. The observed visible-light-assisted photoresponse may therefore involve defect- or interface-related electronic states, optical absorption associated with the rGO-containing composite, and possible MB photosensitization. The π-conjugated rGO network may act as a conductive charge-transfer platform capable of accepting and transporting photoinduced electrons, thereby reducing electron–hole recombination losses.^17,22,25,33^

If photoinduced carriers are populated in the electronic states of ZnO and Zn_2_SnO_4_ through one or more of these excitation processes, their subsequent interfacial migration may conceptually follow Type-II-like or Z-scheme-like pathways. In a Type-II-like pathway, electrons and holes would migrate toward energetically favorable band-edge states in the two semiconductors, promoting spatial charge separation but potentially reducing the redox ability of the accumulated carriers. By contrast, a Z-scheme-like pathway would involve interfacial recombination of the relatively weaker carriers while retaining electrons and holes with stronger reduction and oxidation capabilities, respectively, potentially favoring ROS formation.^16,23,40^ These pathways describe possible post-excitation interfacial carrier-transfer processes and do not imply direct band-to-band excitation of both oxides by visible light.

The lower PL intensity and higher transient photocurrent response of ZSO2.5 support improved carrier separation and transport following rGO incorporation. However, these measurements do not determine the absolute band-edge positions, the initial excitation processes, or the direction of interfacial carrier migration. Moreover, the substantial contribution of ˙O_2_^−^ indicated by the scavenger experiments supports the involvement of reduction-active electrons but does not, by itself, distinguish between Type-II-like and Z-scheme-like pathways without reliable band-edge positions and direct carrier-tracking evidence. Conclusive identification of the dominant charge-transfer pathway would require complementary evidence from techniques such as VB-XPS or UPS combined with Mott–Schottky analysis, light-irradiated *in situ* XPS, surface photovoltage spectroscopy, EPR spin trapping, selective photodeposition, and theoretical calculations.^16^ Accordingly, the pathways illustrated in Fig. 13(b) should be regarded as plausible conceptual models based on the available experimental observations and literature rather than as experimentally validated charge-transfer mechanisms.

#### 3.11.3. Proposed MB removal mechanism

Besides facilitating carrier transport, rGO may enhance MB adsorption through π–π interactions between its conjugated carbon framework and the aromatic structure of MB.^17,22,25,34^ Electrons transported through rGO may react with dissolved oxygen, provided that they possess sufficient reduction potential:rGO(e^−^) + O_2_ → ˙O_2_^−^

Hydroxyl radicals may be generated through hole-mediated oxidation of surface hydroxyl groups or water, provided that the holes possess sufficient oxidation potential:h^+^ + OH^−^ → ˙OHh^+^ + H_2_O → ˙OH + H^+^

Because the band-edge positions were not determined, these reactions cannot be confirmed solely from the present measurements. Secondary reactions involving oxygen-derived species may also contribute to ˙OH formation.^17,25,36,38^

The generated ˙O_2_^−^, ˙OH, and other reactive species may attack the MB chromophore, leading to oxidative transformation and decolorization:MB + reactive species → transformation products

Possible MB photosensitization may also contribute to electron injection and subsequent ROS generation under visible-light irradiation. Because HPLC, LC-MS, and TOC analyses were not performed, the transformation products and mineralization degree cannot be determined.

Overall, the enhanced MB removal over ZSO2.5 is attributed to the combined contributions of rGO-assisted light absorption and adsorption, interfacial carrier transport, and ROS generation, particularly ˙O_2_^−^. This interpretation is consistent with the UV-vis, PL, photocurrent, and scavenger results, although the detailed carrier-transfer pathway and molecular transformation products require further direct investigation.

## 4. Conclusions

ZSO/rGO ternary composites were successfully synthesized *via* a hydrothermal method. XRD confirmed the coexistence of hexagonal ZnO and cubic spinel Zn_2_SnO_4_, while Raman spectroscopy supported the incorporation of rGO. Electron microscopy revealed oxide particles distributed in close contact with thin rGO sheets. XPS indicated the presence of Zn^2+^, Sn^4+^, lattice and surface-related oxygen species, and an sp^2^-dominated carbon framework. The observed Zn 2p binding-energy shift was consistent with a change in the local electronic environment after Zn_2_SnO_4_ and rGO incorporation.

Among the investigated samples, ZSO2.5, containing 2.5 wt% rGO, exhibited the lowest PL intensity and highest transient photocurrent response. Considered together, these results support reduced radiative recombination and improved photoinduced carrier transport. However, the available measurements do not establish the band-edge positions or direction of carrier migration. Therefore, the interfacial charge-transfer pathway cannot be conclusively assigned as Type-II or Z-scheme-like.

ZSO2.5 achieved the highest MB removal efficiency of 87.94% after 120 min of visible-light irradiation, with an apparent rate constant of 0.01764 min^−1^. Scavenger experiments suggested the qualitative importance order ˙O_2_^−^ > ˙OH > h^+^ among the investigated reactive species. ZSO2.5 also retained comparable activity over three consecutive cycles, demonstrating good short-term reusability, although longer tests and post-cycling characterization are required to establish long-term stability. Because the transformation products and mineralization degree were not determined, the observed process is described as MB removal and decolorization rather than complete degradation or mineralization. Overall, an appropriate rGO content improves the optical response, interfacial carrier transport, and photocatalytic performance of ZSO/rGO composites, supporting their potential for visible-light-assisted water treatment.

## Author contributions

Pham Van Tuan: writing – review & editing, writing – original draft, methodology, investigation, data curation, conceptualization, funding acquisition. Hoang Phi Hung: methodology, investigation. Vu Thi Tam Hieu: investigation, data curation. Tran Ngoc Khiem: investigation, formal analysis. Le Tien Ha: writing – review & editing, methodology, investigation, data curation, conceptualization.

## Conflicts of interest

The authors declare that they have no known competing financial interests or personal relationships that could have appeared to influence the work reported in this paper.

## Supplementary Material

RA-016-D6RA05978C-s001

## Data Availability

All data generated or analyzed during this study are included in this published article. No additional datasets were created or analyzed. Any supplementary inquiries may be directed to the corresponding authors (tuan.phamvan@hust.edu.vn and halt@tnus.edu.vn). Supplementary information (SI) is available. See DOI: https://doi.org/10.1039/d6ra05978c.
