# Supplementary material for: Visible-light-assisted photocatalytic removal of methylene blue over ternary ZnO/Zn2SnO4/rGO heterostructures: structural, optical, and charge-transport behavior
Source: RSC Adv. 2026 Sep 3;16(43):52848–62. doi: 10.1039/d6ra05978c (PMC13541075; doi:10.1039/d6ra05978c)
Supplement: Supplementary file 1 [file RA-016-D6RA05978C-s001.pdf]

Supplementary Information (SI) for RSC Advances.
This journal is © The Royal Society of Chemistry 2026

Supporting information

Visible-Light-Assisted Photocatalytic Removal of Methylene Blue over Ternary $ZnO/Zn_2SnO_4/rGO$ Heterostructures: Structural, Optical, and Charge-Transport Behavior

Pham Van Tuan[1, *], Hoang Phi Hung[1], Vu Thi Tam Hieu[2], Le Tien Ha[2,*], and Tran Ngoc Khiem[1]

[1]School of Materials Science and Engineering, Hanoi University of Science and Technology, No. 1, Dai Co Viet, Hanoi, Vietnam

[2]Institute of Science and Technology, TNU-University of Sciences, Phan Dinh Phung, Thai Nguyen, 250000, Vietnam

Corresponding author's email: halt@tnus.edu.vn; tuan.phamvan@hust.edu.vn

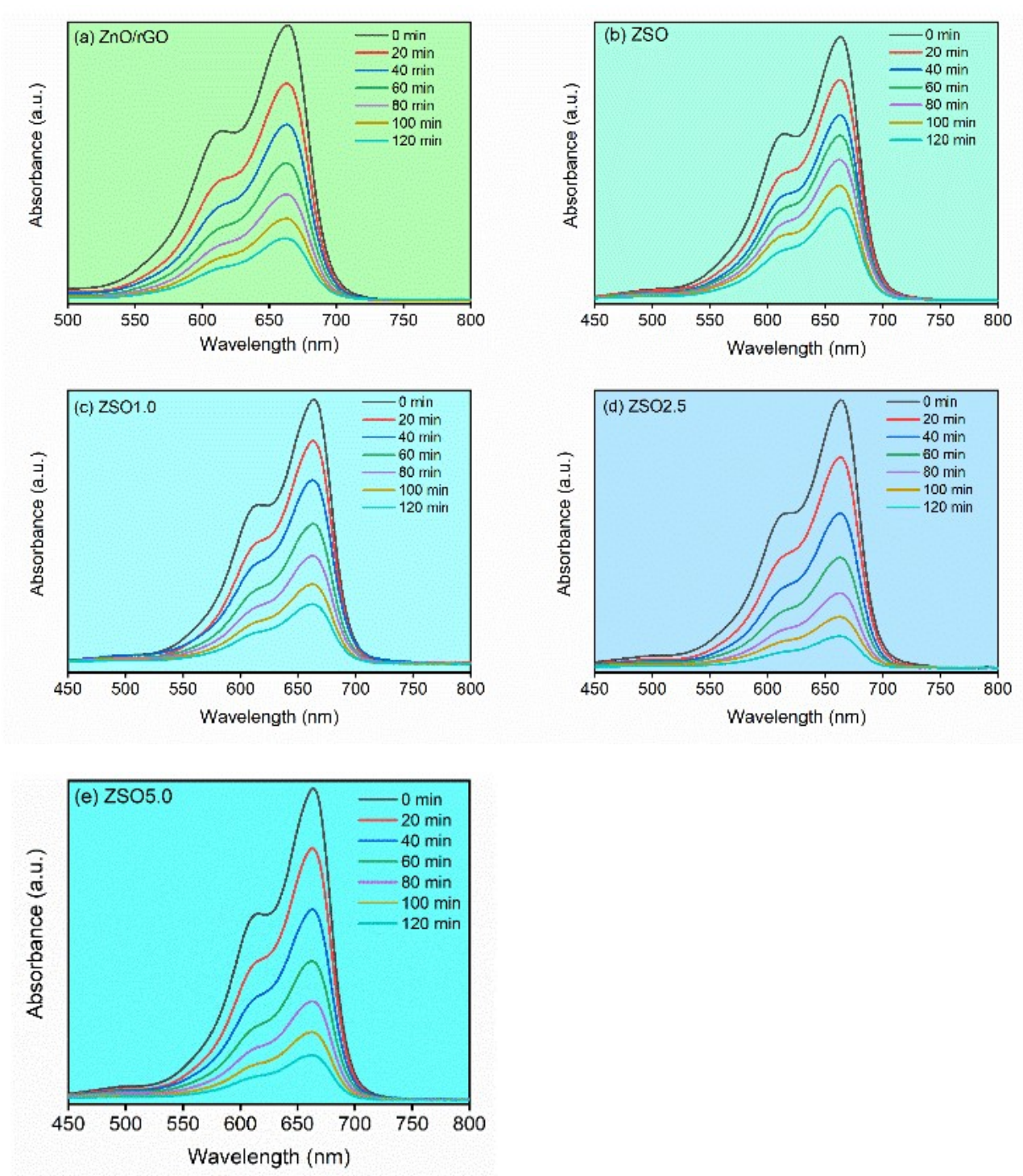

Figure S1. Time-dependent UV–Vis absorption spectra of MB during visible-light-assisted removal using ZnO/rGO, binary ZSO, and ternary ZSO/rGO photocatalysts with different rGO loadings.
